# The nuclear GUCT domain-containing DEAD-box RNA helicases govern gametophytic and sporophytic development in *Physcomitrium patens*

**DOI:** 10.1007/s11103-021-01152-w

**Published:** 2021-04-22

**Authors:** Pierre-François Perroud, Viktor Demko, Ako Eugene Ako, Rajendra Khanal, Boris Bokor, Andrej Pavlovič, Ján Jásik, Wenche Johansen

**Affiliations:** 1 Plant Cell Biology, Faculty of Biology, University of Marburg, Karl-von-Frisch Str. 8, 35043 Marburg, Germany; 2 Department of Plant Physiology, Faculty of Natural Sciences, Comenius University in Bratislava, Ilkovicova 6, 84215 Bratislava, Slovakia; 3 Plant Science and Biodiversity Center, Slovak Academy of Sciences, Dúbravská cesta 9, 84523 Bratislava, Slovakia; 4 Department of Biotechnology, Inland Norway University of Applied Sciences, Holsetgata 31, 2318 Hamar, Norway; 5 Comenius University in Bratislava Science Park, Ilkovicova 8, 84215 Bratislava, Slovakia; 6 Department of Biophysics, Centre of the Region Haná for Biotechnological and Agricultural Research, Faculty of Science, Palacký University, Šlechtitelů 27, 78371 Olomouc, Czech Republic; 7 Present Address: Institut Jean-Pierre Bourgin, INRAE, AgroParisTech, Université Paris-Saclay, 78000 Versailles, France; 8 Present Address: School of Animal, Rural and Environmental Sciences, Nottingham Trent University, Brackenhurst Campus, Southwell, NG25 0QF Nottinghamshire UK; 9 Present Address: Department of Gastroenterology, Hepatology and Endocrinology, Hannover Medical School, Carl-Neuberg-Str. 1, 30625 Hannover, Germany

**Keywords:** RNA helicase, Development, *Physcomitrium patens*, Gametophyte, Sporophyte, Starch accumulation

## Abstract

**Key message:**

In *Physcomitrium patens, Pp*RH1/*Pp*RH2 are GUCT-domain-containing DEAD-BOX RNA helicases localize to the nucleus. They are implicated in cell and tissue development in all stages of the moss life cycle.

**Abstract:**

The DEAD-box-containing RNA helicase family encompasses a large and functionally important group of enzymes involved in cellular processes committed to the metabolism of RNA, including its transcription, processing, transport, translation and decay. Studies indicate this protein family has implied roles in plant vegetative and reproductive developmental processes as well as response to environmental stresses such has cold and high salinity. We focus here on a small conserved sub-group of GUCT domain-containing RNA helicase in the moss *Physcomitrium patens.* Phylogenetic analysis shows that RNA helicases containing the GUCT domain form a distinct conserved clade across the green lineage. In this clade, the *P. patens* genome possesses two closely related paralogues RNA helicases predicted to be nuclear, *PpRH1* and *PpRH2*. Using in-locus gene fluorescent tagging we show that *Pp*RH1 is localized to the nucleus in protonema. Analysis of *PpRH1* and *PpRH2* deletions, individually and together, indicates their potential roles in protonema, gametophore and sporophyte cellular and tissue development in *P. patens*. Additionally, the ultrastructural analysis of phyllid chloroplasts in *Δrh2* and *Δrh1/*2 shows distinct starch granule accumulation under standard growth conditions associated with changes in photosynthetic activity parameters. We could not detect effects of either temperature or stress on protonema growth or *PpRH1* and *PpRH2* expression*.* Together, these results suggest that nuclear GUCT-containing RNA helicases play a role primarily in developmental processes directly or indirectly linked to photosynthesis activity in the moss *P. patens.*

**Supplementary Information:**

The online version contains supplementary material available at 10.1007/s11103-021-01152-w.

## Introduction

RNA helicases are important participants in essentially all biological processes related to RNA metabolism. These enzymes function as molecular motors to remodel RNA structures and ribonucleoprotein (RPN) complexes at the expense of adenosine triphosphate (ATP) hydrolysis in housekeeping pathways such as processing and editing of nuclear, chloroplast and mitochondrial transcripts, export and degradation of mRNA, translation initiation and ribosome biogenesis (Tanner and Linder 2001). Based on comparative structural and functional analyses, sequence similarities and conservation of specific motifs within the catalytic core of the enzyme, RNA helicases are currently classified into six superfamilies (SF1–SF6) (Singleton et al. 2007). SF2, the largest of the superfamilies, is further divided into several defined families, one of which is the DEAD-box family of RNA helicases (Fairman-Williams et al. 2010). The proteins in this family have received their name from the presence of a conserved stretch of amino acids D-E-A-D (Asp-Glu-Ala-Asp) in motif II. Motif II is one of 13 described conserved motifs (Q, I, Ia, Ib, Ic, II, III, IV, IVa, V, Va, Vb and VI) defining the RNA helicases in this family (Fairman-Williams et al. 2010). The conserved motifs, required for ATP and RNA binding and for linking phosphoanhydride cleavage of ATP with helicase activity are distributed over two similar RecA-like domains, arranged in tandem and tethered by a flexible linker, constituting the basic catalytic helicase core (Tanner and Linder 2001). The eukaryotic eIF4A-family translation initiation factors, the smallest and founding members of DEAD-box RNA helicase family, consist of an isolated helicase core without N- and C-terminal extensions/domains (Andreou and Klostermeier 2014), which are typically present in the majority of the DEAD-box proteins. These auxiliary extensions and domains mediate interactions with ATP, RNA and protein partners and are proposed to provide signals for subcellular localization, for modulating the activity of the helicase core and are thought to be critical for the physiological specificity of the helicase, thereby contributing to the functional diversity of this protein family (Fairman-Williams et al. 2010). The basic biochemical reaction of DEAD-box proteins, RNA unwinding coupled to ATPase activity, is just one of several activities identified for these enzymes; some enzymes display strand annealing activity, promoting duplex formation, others are involved in protein displacement to remodel RPN complexes or function as assembly platforms to increase their size (Putnam and Jankowsky 2013).

The RNA helicase gene families in plants are larger and more diverse compared to those in other organisms (Linder and Owttrim 2009). The analysis of the complete genomes from *Arabidopsis thaliana*, *Oryza sativa*, *Glycine max* and *Zea mays* revealed 161, 149, 136 and 213 unique genes, respectively, encoding putative RNA helicases in these organisms (Xu et al. 2013). Of these, 50, 51, 57 and 87 proteins were classified as DEAD-box RNA helicases, respectively. Experimental evidence for biochemical activities of these proteins is limited, but available data shows that plant DEAD-box RNA helicases have important functions in plant growth and development, and in various stress responses (Linder and Owttrim 2009). eIF4A proteins are among the more thoroughly characterized DEAD-box proteins. Plants (and mammalians) express three eIF4A proteins, eIF4A1, eIF4A2 and eIF4A3, exhibiting RNA-stimulated ATPase activity and RNA unwinding activity (Rogers et al. 1999), functioning in translation initiation (eIF4A1 and eIF4A2) and in the assembly of the exon junction complex (eIF4A3) (reviewed by Andreou and Klostermeier 2014). Available data from several plant species clearly indicate that elF4A proteins are essential for normal plant growth and development, possibly by regulating protein translation during the cell cycle (Hutchins et al. 2004; Bush et al. 2009, 2016). In *A. thaliana*, both eIF4A1 (AT3G13920) and eIF4A2 (AT1G54270) are expressed in actively dividing cells, and an elF4A1 mutant, producing a putative truncated elF4A1 version, shows slow growth and reduced lateral root formation, delayed flowering and abnormal ovule development (Bush et al. 2015). In the early land plant *Physcomitrium patens*, deletion of a conserved region of *Pp3c6_1080V3.1*, encoding the helicase core motifs of eIF4A1/eIF4A2, produce mutant plants with slowed protonemata growth and dwarfed leafy gametophores with reduced stems and internodes, suggesting a role for eIF4A1/eIF4A2 in cell division and/or elongation (Tyagi et al. 2020). In the same study, a function for eIF4A1/eIF4A2 in stress response was identified by the observation of a strong and rapid accumulation of *Pp3c6_1080V3.1* transcripts under increased salinity. The *Pp3c6_1080V3.1* knock-out mutant exhibits a slow-recovery phenotype after salt stress treatment, also suggesting a role for eIF4A1/eIF4A2 in stress management in *P. patens*. Several other DEAD-box proteins have been shown to be involved in various stress responses in plants. The DEAD-box protein TOGR1 (Thermotolerant Growth Required1; LOC_Os03g46610) in *O. sativa* maintains normal rRNA homeostasis at high temperature, the helicase activity directly promoted by an increase in temperature (Wang et al. 2016). Conversely, DEAD-box RNA helicase 42 (OsRH42, LOC_Os08g06344) in *O. sativa* was shown to be necessary for effective splicing of pre-mRNA during mRNA maturation at low temperatures (Lu et al. 2020). In two comprehensive expression profiling studies in tomato, organ- and/or tissue-specific expression patterns of DEAD-box RNA helicases were observed in relation to various abiotic (Cai et al. 2018) and biotic (Pandey et al. 2019) stress conditions, thus providing evidence for RNA helicases performing diverse roles in cellular stress responses and during plant growth and development.

DEAD-box RNA helicases function in different compartments of the cell, including the cytosol, nucleus, chloroplast and mitochondria. *A. thaliana* DEAD-box RNA helicase 7 (AtRH7/PRH75; AT5G62190) is localized to the nucleolus (Lorković et al. 1997), harbours dsRNA unwinding activity (Nayak et al. 2013) and participates in pre-rRNA processing and possibly ribosome assembly (Huang et al. 2016). *AtRH7* is ubiquitously expressed in both vegetative and reproductive tissue and promoter-GUS constructs indicated that this gene is transcriptionally active mainly in regions undergoing cell division (Huang et al. 2016). Disruption of the *AtRH7* gene caused severe developmental defects including abnormal embryo and seedling development, in addition to delayed seed germination showing the importance of this protein in plant development (Huang et al. 2016). *AtRH7* is also implicated in cold tolerance/acclimation as *AtRH7* transcription is slightly upregulated in cold-treated seedlings; transcript levels are unaffected by osmotic, salt, drought and heat stresses. AtRH3 (AT5G26742), DEAD-box RNA helicase 3 from *A. thaliana*, is localized to the chloroplasts (Peltier et al. 2006). The accumulation of AtRH3 and the maize orthologue (ZmRH3A) are strongly controlled during plant development, the RH3 proteins functioning in splicing of group II introns, maturation of rRNA and possible also in ribosome biogenesis (Asakura et al. 2012). The *AtRH3* gene is essential in *A. thaliana* as null mutants are embryo lethal. AtRH3 and AtRH7 both contain a C-terminal GUCT (NUC152) domain for which no function has been described. A GUCT domain has also been identified in human RNA DEAD-box protein 21 (DDX50; RHII/Guβ), a multifunctional enzyme with RNA-unwinding and RNA-folding activities (Valdez et al. 1997). Bioinformatic analyses of the GUCT domain have shown the presence of the RNA recognition motif (RRM) fold in RH-II/Guβ, a fold typically found in RNA-interacting proteins, however without the essential amino acid residues important for RNA recognition, suggesting that the GUCT domain of RH-II/Guβ is unlikely to interact with RNA (Ohnishi et al. 2009). Mutagenesis studies also showed that the GUCT domain is not essential for RH-II/Guβ helicase or foldase activity (Valdez et al. 1997; Valdez 2000). Thus, the function of the GUCT domain in DEAD-box RNA helicases still remains elusive.

With diverse domain composition and sub-cellular localization, DEAD-box RNA helicases have been implicated in a broad array of developmental and stress-related functions. Our research aims to elucidate the biological role of two newly identified GUCT-domain containing DEAD-box RNA helicases (*PpRH1*; Pp3c20_20710 and *PpRH2*; Pp3c10_20840) in the moss *P. patens*. Using primarily a reverse genetic approach, we focus on phenotypic characterization to pinpoint their function in both developmental processes and stress responses. In the present study, we show that the GUCT-domain containing DEAD-box RNA helicases are present in the green lineage since its inception. We demonstrate *Pp*RH1 nuclear localization in the moss *P. patens*. Through the individual deletion of *PpRH1* and *PpRH2* and their deletion in a double mutant, we show that, together, they are necessary for normal morphological vegetative development and necessary for sporophytic development. In contrast to other plant DEAD-box RNA helicases, GUCT-domain-containing DEAD-box RNA helicases apparently do not affect moss response to environmental stresses but *PpRH1* and *PpRH2* mutants display both aberrant photosynthetic activity and starch accumulation.

## Results

### PpRNA helicase 1 and 2 belong to the DEAD-box RNA helicase family

**Fig. 1:**
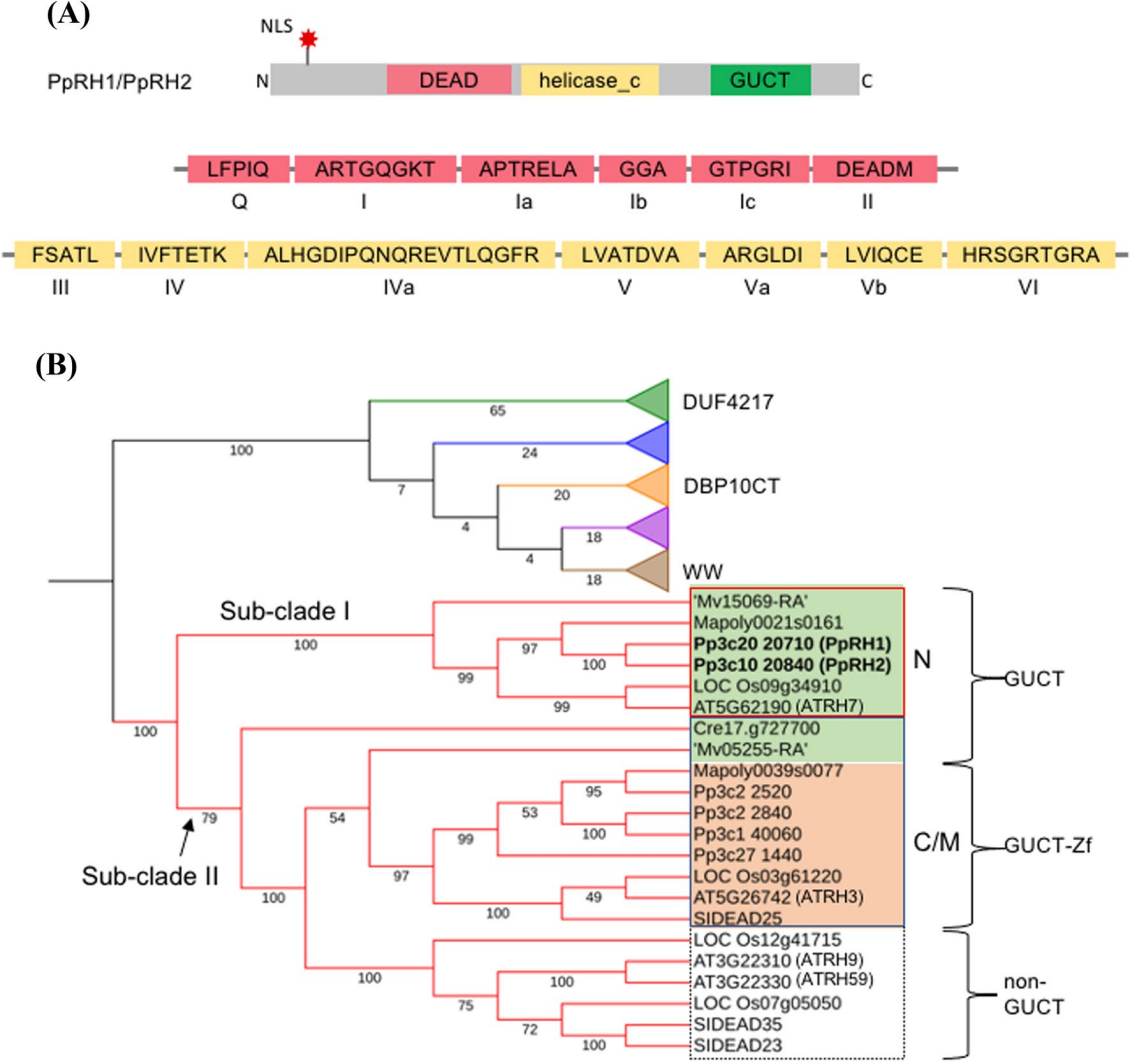
Structure and phylogenetic analysis of *Pp*RH1 and *Pp*RH2. **a** Schematic representation of the identical domain structure (DEAD (red), helicase_c (yellow) and GUCT (green), core motifs (Q, I, Ia, Ib, Ic, II, III, IV, IVa, V, Va, Vb and VI) and the predicted nuclear localization signal (NLS) (red star) in proteins *Pp*RH1 and *Pp*RH2. The amino acid sequence of the motifs is highlighted in the red and yellow boxes and are identical between the two proteins. Figure not drawn to scale. **b** Partly collapsed phylogenetic tree of the DEAD-box RNA helicase family members from *A. thaliana*, *S. lycopersicum, O. sativa*, *P. patens*, *M. polymorpha*, *M. viride* and *C. reinhardtii*. All the GUCT-containing helicases are clustered into one major clade, separated from the majority of the remainder of the RNA helicases which are further clustered into 5 subclades. The helicases containing the DUF4217, DBP10CT or WW domains are each clustered separately into individual sub-clades as shown in more detail in Supplementary Fig. S1. The helicases containing GUCT (green box) or GUCT-Zf_CCHC (orange box) are clustered into two sub-clades, I (red frame) and II (blue frame), and are predicted and/or annotated to be localized to the nucleus (N) or the chloroplast/mitochondria (C/M), respectively. The non-GUCT containing RNA helicases are shown in in the white box

The *P. patens* RNA helicase 1 (Pp3c20_20710) and RNA helicase 2 (Pp3c10_20840), hereafter referred to as *Pp*RH1 and *Pp*RH2, respectively, are 87% identical at the amino acid level and are encoded by 7-exon-genes on chromosomes 20 and 10, respectively. While the exon structure of the corresponding genes is highly conserved, their coding sequences being 79% identical, the sizes of the introns vary between the two, particularly intron 1 of *PpRH2*, which is 46% larger than that of *PpRH1* (Supplementary Table S1). The two proteins contain putative nuclear localization signals (NLS), a conserved helicase core region with the DEAD-box motif and consisting of the Pfam domains DEAD (Pfam code: PF00270) and helicase_c (Pfam code: PF00271), in addition to a C-terminal GUCT domain (Pfam code: PF08152) (Fig. 1a). The 13 described signature motifs characteristic for the helicase core of SF2 proteins, Q, I, Ia, Ib, Ic, II, III, IV, IVa, V, Va, Vb and VI (Fairman-Williams et al. 2010) are present in both *Pp*RH1 and *Pp*RH2, and are 100% conserved between the two proteins (Fig. 1a). Of the three domains identified, GUCT is the less conserved domain of *Pp*RH1 and *Pp*RH2 with a sequence identity of 86%. GUCT domains are currently identified in approximately 1000 different Pfam sequences, arranged in 30 different protein architectures, mainly in combination with the DEAD and/or helicase_c domains of eukaryotic proteins. Interestingly, the GUCT domain has so far not been identified in archea, viruses and viroids, and only in a limited number (23) of bacterial species exclusively belonging to the *Deinococcus-Thermus* phylum, a group of extremophiles known to be highly resistant to environmental hazards, including radiation and high/low temperatures (Ho et al. 2016).

Sequence similarity searches (pBLAST) using *Pp*RH1 as query resulted in the identification of 47 *P. patens*, 28 *Marchantia polymorpha,* 35 *Mesostigma viride* and 37 *Chlamydomonas reinhardtii* RNA helicases containing the DEAD-box motif. The domain structure and sizes of these proteins, in addition to their homologues in *Solanum lycopersicum* (Pandey et al. 2019), *O. sativa* and *A. thaliana* (Xu et al. 2013) can be found in Supplementary Table S2. Structural analysis of the selected RNA helicases from these organisms, representing major *Viridiplantae* groups, revealed that only a restricted number of additional domains, including WW (PF00397), DUF4217 (PF13959), DBP10CT (PF08147), GUCT and Zf_CCHC (PF00098), are found in combination and in an identical arrangement to the helicase core. With the exception of zinc knuckle (Zf_CCHC) domain, all other domains (WW, DUF4217, DBP10CT, GUCT) were found across all analyzed species, suggesting that the different DEAD-box RNA helicase structures were established already in the common ancestor of Chlorophyta and Streptophyta. To further investigate the evolutionary relationship of the sampled DEAD-box RNA helicase, a phylogenetic analysis was performed using the core helicase region of the proteins listed in Supplementary Table S2. In this analysis, we excluded sequences containing large insertions between the two helicase core domains (DEAD and helicase_c), a specific architecture we exclusively detected in the algae, possibly representing an evolutionary group lost by land plants. Similar to previous studies (Pandey et al. 2019), the phylogenetic analysis revealed that the DEAD-box RNA helicases cluster into six clades (Fig. 1b and Supplementary Fig. S1). *Pp*RH1 and *Pp*RH2 form one major phylogenetic group together with all RNA helicases containing the GUCT domain, distinctly separated from the vast majority of the other DEAD-box RNA helicases, which are further clustered into five sub-clades. RNA helicases containing the WW, DUF4217 or DBP10CT domains are each clustered separately into one sub-clade (Fig. 1b and Supplementary Fig. S1). The clade containing the RNA helicases harboring the GUCT domain can be further divided into two sub-clades (Fig. 1b); one sub-clade (I) consists of proteins exclusively with the DEAD-helicase_C-GUCT domain architecture and the second sub-clade (II) contains RNA helicases with either the helicase core only, the helicase core plus GUCT or the helicase core with the GUCT and Zf_CCHC domains arranged in tandem. Importantly, all RNA helicases in sub-clade I are predicted to be nuclear localized, as opposed to the GUCT-Zf_CCHC-containing RNA helicases in sub-clade II which are either predicted or annotated as chloroplastic or mitochondrial proteins (Supplementary Table S2). In agreement with the prediction of a nuclear localization signal, the *A. thaliana* DEAD-box ATP-dependent RNA helicase 7 (also known as AtRH7; AT5G62190) was previously shown experimentally to be localized to the nucleus (Lorković et al. 1997). This evidence supports our phylogenetic analysis suggesting that GUCT-containing RNA helicases are clearly organelle associated proteins, destined either for the nucleus or the mitochondria/chloroplast. Our results further suggest that the Zf_CCHC domain of the GUCT-containing RNA helicases was established in land plants after the split of the charophyte algae.

### PpRH1 and PpRH2 are ubiquitously expressed in *P. patens* tissues

**Fig. 2:**
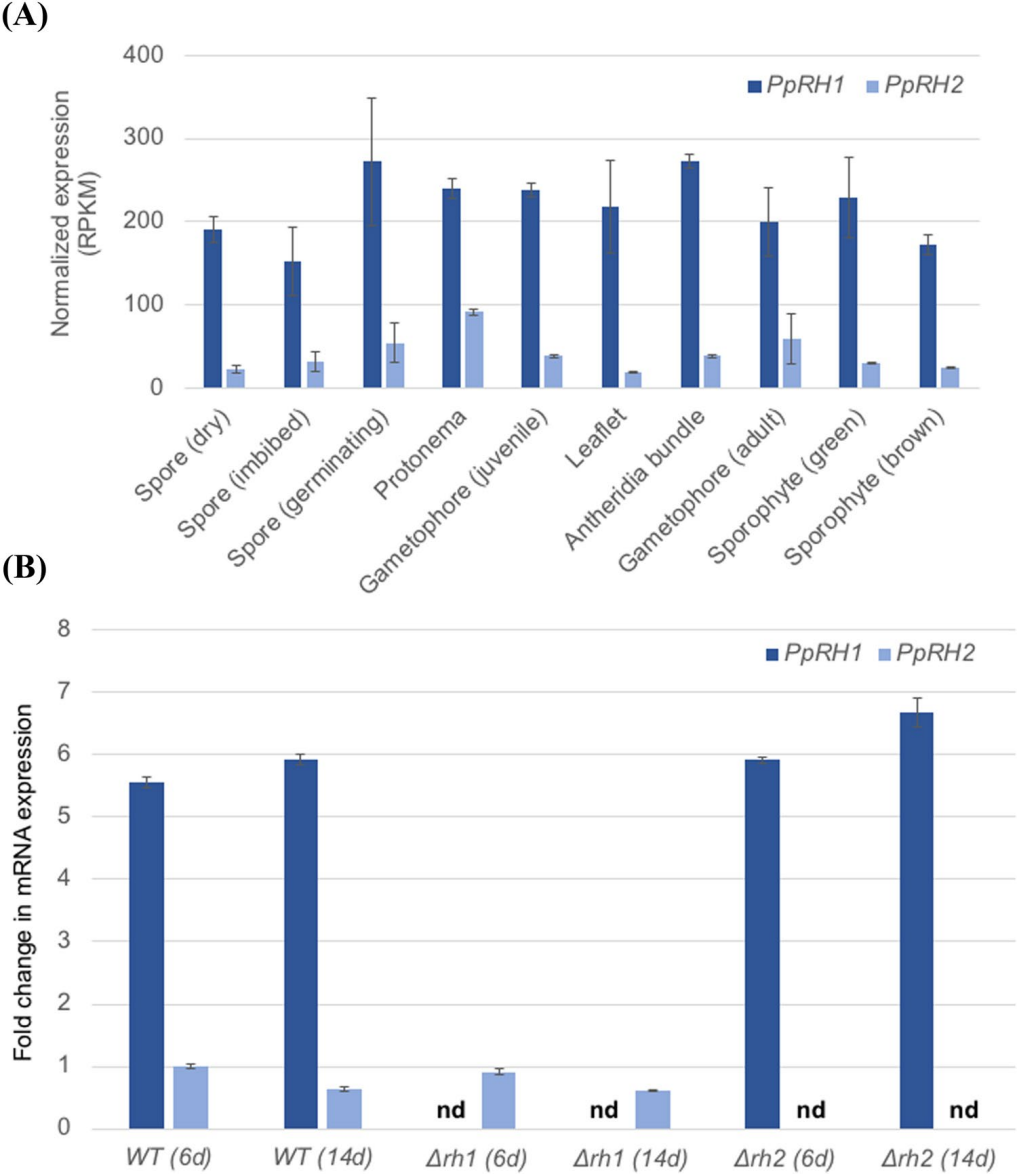
*PpRH1* and *PpRH2* transcript accumulation in *Physcomitrium patens* wild-type and mutant plants. **a** Bar plots showing the normalised expression levels (in RPKM) of *PpRH1* and *PpRH2* at different developmental stages of the WT *P. patens* life cycle. Data extracted from the RNA-seq Developmental stages gmv3.3 dataset of the PEATmoss online tool and from the Meyberg et al. (2020) RNA-seq experiment for the antheridia bundles stage. **b** Quantitative reverse-transcription PCR analysis representing fold change in *PpRH1* and *PpRH2* gene expression in wild-type and mutant plants (*Δrh1* and *Δrh2*) grown for 6 and 14 days relative to the *PpRH2* level in 6-day-old wild-type plants, which was set to 1. Error bars represent the standard error (se) of two biological replicates. nd: no detectable transcripts; d: days

We performed a developmental transcript accumulation profiling using the RNA-seq Developmental stages gmv3.3 data set and the PEATmoss tool (Fernandez-Pozo et al. 2020). Both *PpRH1* and *PpRH2* appear to be ubiquitously expressed throughout the *P. patens* life cycle (Fig. 2a). However, *PpRH1* is systematically expressed 2 to 10 times higher than *PpRH2* across different tissues. This expression pattern was independently observed by the *Physcomitrella* eFP browser tool (Ortiz-Ramírez et al. 2016) (Supplementary Fig. S2). Subsequently quantitative reverse-transcription polymerase chain reaction (qRT-PCR) performed on protonema after 6 days of growth and protonema with gametophores after 14 days of growth confirmed the clear differential expression between *PpRH1* and *PpRH2* (Fig. 2b)*.*

According to the information extracted from the PEATmoss tool, variations of environmental conditions such as light, temperature or the hormone abscisic acid (ABA) do not appear to have an effect on the transcription of *PpRH1* and *PpRH2* (data not shown). Similarly, other studies did not detect any variation in transcript accumulation from these two genes in response to either mild or high heat (Elzanati et al. 2020) or cold treatment (Khraiwesh et al. 2015).

### PpRH1 is localized in the nucleus of growing protonema

**Fig. 3:**
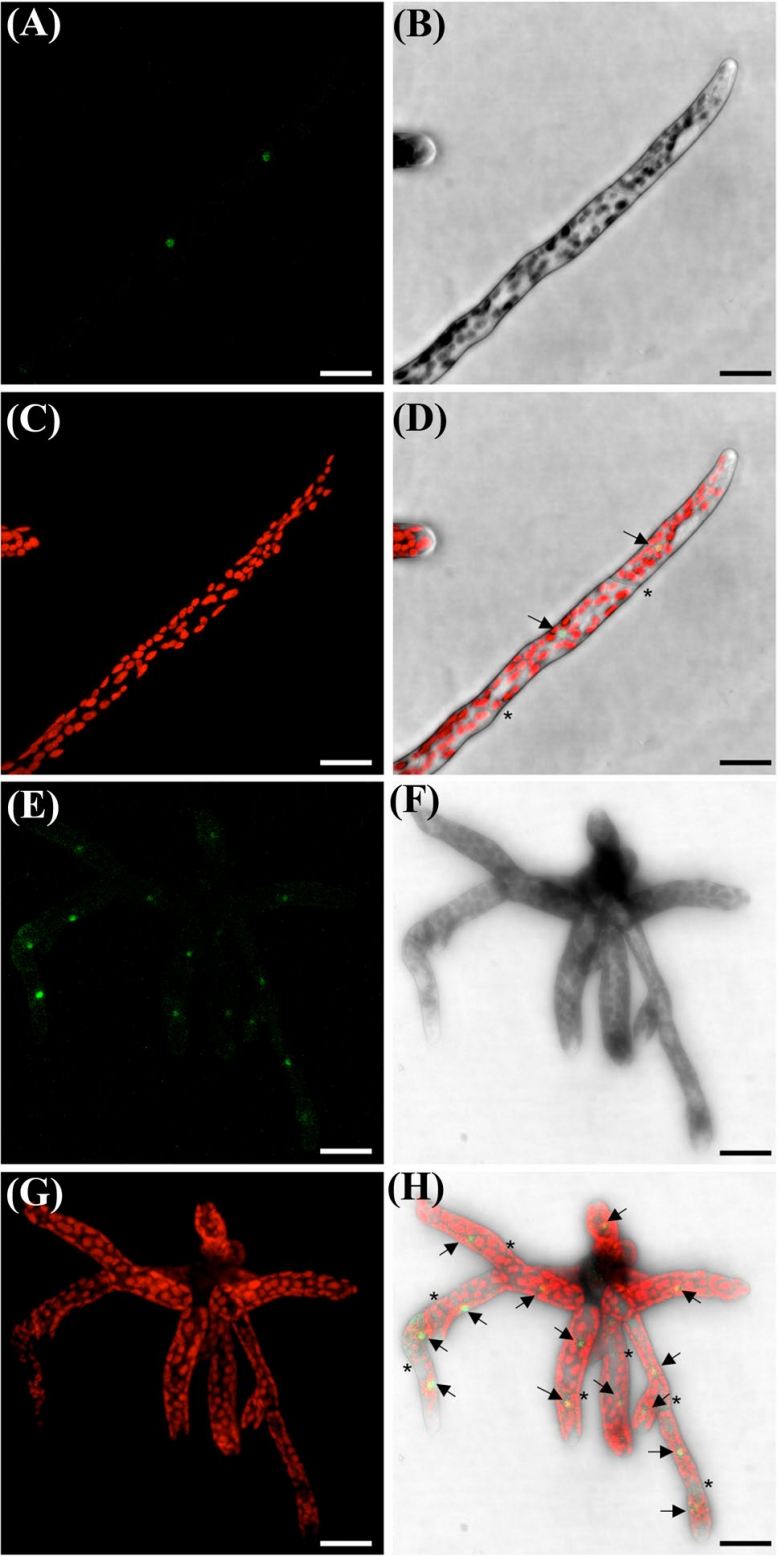
*Pp*RH1-3xGFP localizes in the nucleus of *Physcomitrium patens* protonemal cells. **a–d** Tip cell of filament propagated vegetatively. Bar: 25 μm. **e–h** Five-day-old sporeling. *Pp*RH1-3xGFP specific fluorescent signal confocal stack. **b** and **f** Average projection of confocal stack of transmitted light signal. **c** and **g** Maximum projection of chlorophyll auto-fluorescence signal confocal stack. **d** and **h** Merged **a–c** and **e**–**f** images, respectively. Arrows point to nuclei. * denotes the filament intercalary cell walls. Bar: 25 μm

To evaluate the cellular localization of *Pp*RH1, we generated the *rh1-3xgfp* strain containing a GFP triplicate C-terminal tag (Supplementary Result S1 and Supplementary Fig. S3). *rh1-3xgfp* is morphologically undistinguishable from wild-type (WT) during the gametophytic and sporophytic phases of its life cycle. This strain displays a low GFP signal only detectable using a time-gating approach to subtract non-specific background fluorescence (Perroud et al. 2020). *Rhl1-3xgfp* shows a nuclear-specific signal in protonemal cells of blended cultures of the primary transformant (Fig. 3a–c and Supplementary Fig. S4 for comparison with untransformed control) as well as in young sporeling (Fig. 3d–f) produced by selfed sporophytes. The fluorescent signal is strongest in cells near the tip and nuclear signal intensity is reducing along the filament main branch. In secondary branching filaments, this pattern is repeated. No fluorescent signal, nuclear or otherwise, was detected in other vegetative, sexual or sporophytic tissues.

### The lack of PpRH1 and PpRH2 in single and double mutants differently affects gametophyte growth and development

**Fig. 4:**
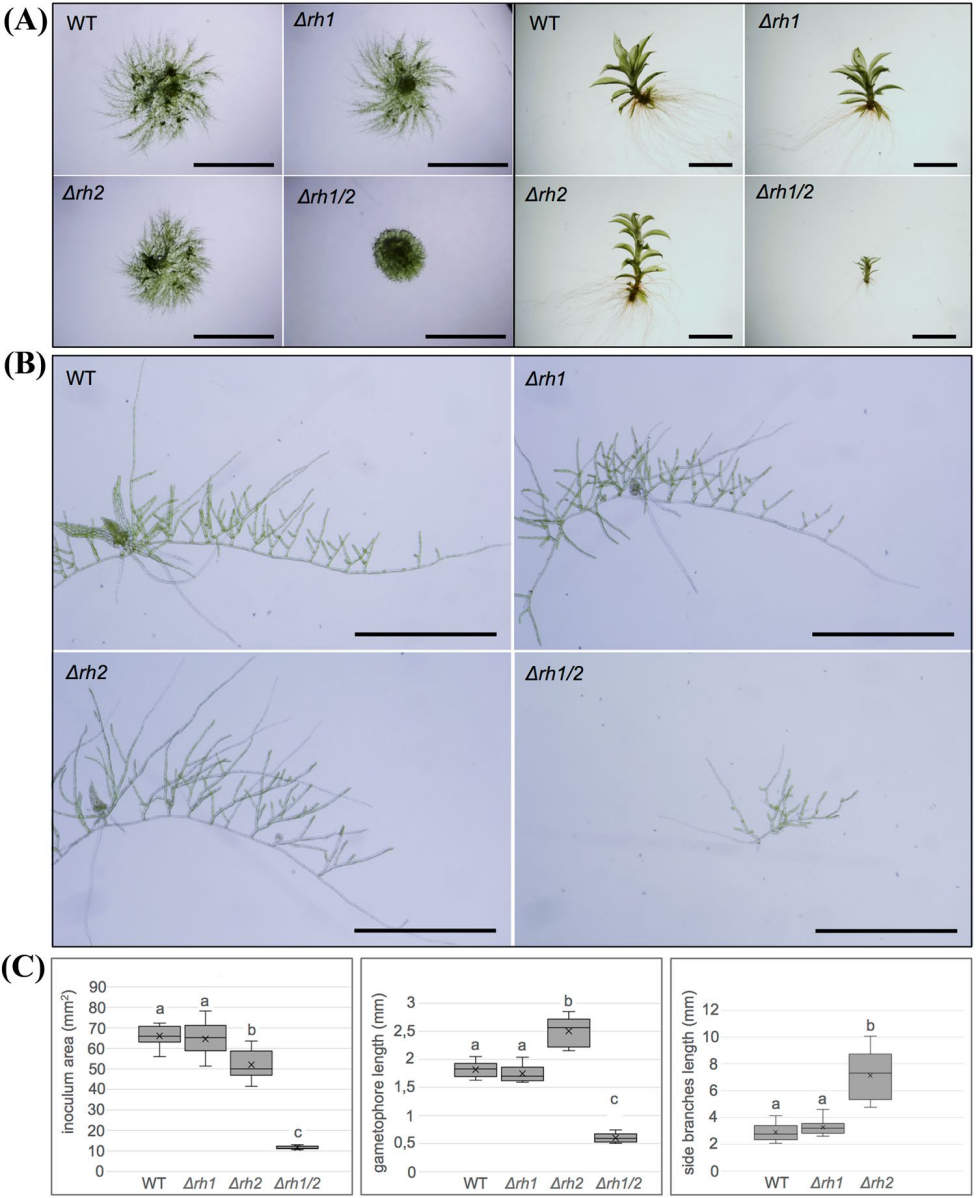
Gametophyte growth of WT, *Δrh1, Δrh2* and *Δrh1/2* under the standard conditions. **a** Two-week-old spot inocula grown on minimal BCD medium (left panel) and isolated one-month-old gametophores (right panel). **b** Detailed view of isolated filaments showing accelerated protonemata side branch growth in *Δrh2* and aberrant protonemata development in *Δrh1/2* compared to WT and *Δrh1*. **c** Left side graph: the area of ellipse surrounding 2-week-old inocula from individual strains as depicted on **a**, Middle graph: the length of gametophores measured from the base to the apex of stems. Right side graph: the summed length of first ten side branches counted from the tip of the primary filament. Measurements were performed using PROMICRA QuickPHOTO MICRO 3.0 software. For each strain, twelve plants/gametophores/filaments taken from four independent cultures were measured (n = 12). Statistical significance (**a–c**) of the means was assessed at 95% confidence. Analysis of variance (ANOVA) and least significant difference (LSD) was performed in multiple sample comparison. Scale bars: 5,0 mm in **a** left panel, 2.0 mm in a right panel, 1.0 mm in (**b**)

To study the function of *Pp*RH1 and *Pp*RH2, we generated the single gene knockout lines *Δrh1* and *Δrh2,* respectively. In addition, we generated the double knockout line *Δrh1/2*, in which both *PpRH1* and *PpRH2* were deleted (for information regarding the mutants we refer to Supplementary Result S1 and Supplementary Fig. S5). We first assessed the role of *Pp*RH1 and *Pp*RH2 during gametophytic development of *P. patens* by analyzing *Δrh1*, *Δrh2* and *Δrh1/2* alongside with wild-type strain (WT) grown under standard conditions (Fig. 4). We did not observe any phenotypic differences between WT and *Δrh1* at any gametophytic growth stage investigated. The *Δrh1* showed wild-type spread of protonemata and wild-type morphology of leafy gametophores (Fig. 4a). The growth of side-branches that extend from primary filaments was also similar between WT and *Δrh1* (Fig. 4b). Quantification of plants spreading, gametophore length and side-branch extension confirmed no significant differences between WT and *Δrh1* (Fig. 4c).

In contrast to *Δrh1*, the phenotypes of *Δrh2* and *Δrh1/2* were strikingly different. The *Δrh2* mutant showed slightly reduced spread of protonemata, however the cultures appeared bushier (Fig. 4a). Closer examination of isolated filaments revealed that increased density of *Δrh2* plants was caused by accelerated growth of side-branches formed on primary filaments (Fig. 4b). Quantitative data showed about three-times longer side branches in *Δrh2* compared to WT and *Δrh1* (Fig. 4c). Gametophores of *Δrh2* mutant were longer compared to WT (Fig. 4a, c), and appeared narrower, resembling signs of mild etiolation, despite growing under standard conditions. This feature of gametophore growth was clearly manifested after long-term cultivation under reduced light (Supplementary Fig. S6).

The deletion of both *PpRH1* and *PpRH2* genes in the *Δrh1/2 mutant* caused a more severe phenotype in protonemata and gametophores. Protonema spreading was strongly reduced resulting in small dense plants (Fig. 4a). Closer examination of isolated filaments revealed an abnormal branching pattern with predominance of chloronemal cells (Fig. 4b). Although with approximately corresponding phyllid numbers, one-month old gametophores of *Δrh1/2* were dramatically stunted in comparison to WT and the single mutants (Fig. 4a, c).

### Stress conditions do not alter developmental differences between mutants

In order to examine whether *Pp*RH1 and *Pp*RH2 contribute to developmental plasticity in response to stress, we cultivated both single mutants and the double mutant for 2 weeks under low light, high light and salt stress. As shown in Supplementary Figs. S7, S8 and S9, both single mutants and double mutant respond to the stress factors similarly to WT while maintaining their characteristic phenotypic features as described above*.* For details, please see Supplementary Result S1.

To assess steady-state levels of *PpRH1* and *PpRH2* transcripts in response to stress treatments, we treated 2-week-old cultures grown under standard conditions with cold (10 °C) and abscisic acid (ABA 10 μM), respectively, for 24 h. The ABA treatment caused mild increase of *PpRH1* expression both in WT and the *Δrh2* mutant, whereas *PpRH2* expression, whose expression was concistently lower than that of *PpRH1*, was not significantly altered in WT nor in *Δrh1* compared to the corresponding un-treated plants (Supplementary Fig. S10). Cold treatment did not affect *PpRH1* expression in WT plants and we measured only a mild transcript increase for this gene in *Δrh2*. No major effect of cold was detected in the expression of *PpRH2* in WT and the *Δrh1* mutant (Supplementary Fig. S10).

### Photosynthetic performance is moderately affected in *Δrh2* and *Δrh1/2* mutants and reflects developmental alterations

**Fig. 5:**
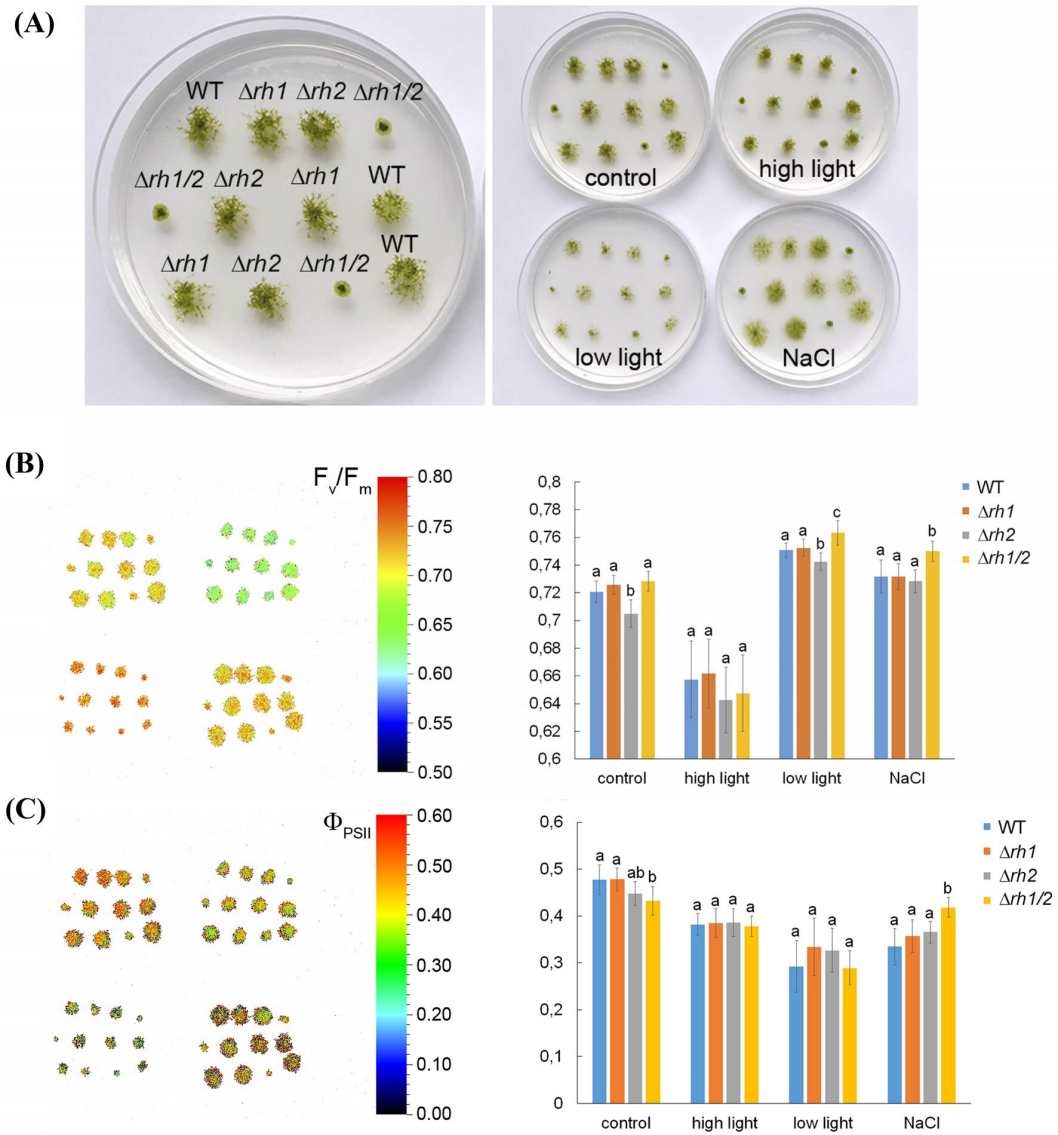
Chlorophyll fluorescence imaging and quantification. **a** Plant distribution on Petri dish (left) and Petri dishes with individual treatments. **b** Maximum quantum yield of PSII (Fv/Fm). **c** Effective photochemical quantum yield of PSII (Φ_PSII_). Representative images with scales on the left in **b** and **c** match plant distribution in four plates/conditions as shown in **a**, on the right, mean values ± SD (n = 12) are shown. For each strain and condition, twelve plants from four independent cultures were measured. Different letters indicate significant differences at *P* < 0.05 within individual treatment (ANOVA, Tukey-test)

Morphological alterations in *PpRH1* and *PpRH2* mutants, and especially etiolation-reminiscent phenotype of gametophores in *Δrh2*, led us to evaluate their photosynthetic performance under standard conditions and when subjected to stress (Fig. 5a).

Maximum quantum yield of photosystem II (F_v_/F_m_) reflects potential quantum efficiency of PSII and is a sensitive indicator of plant photosynthetic performance with optimal values of around 0.83 for most plant species (Maxwell and Johnson 2000). The Bryophytes have lower F_v_/F_m_ values which do not exceed 0.8 indicating the presence of more intensive photoinhibition than in vascular plants at moderate light intensities (Hájek et al. 2009). This was also the case in our study, the highest F_v_/F_m_ values were found under low light condition and the lowest under high-light condition, indicating mild photoinhibition. The *Δrh2* mutant was more sensitive to photoinhibition or showed some other defect in PSII when grown under control and low light conditions in comparison to the *Δrh1 and Δrh1/2* mutants. On the other hand, the *Δrh1/2* double mutant was more resistant to photoinhibition with the highest F_v_/F_m_ values under low light and salt stress conditions (Fig. 5b).

The effective photochemical quantum yield (Φ_PSII_) measures the proportion of light absorbed by chlorophylls associated with PSII that is used in photochemistry and it can provide a measure of the rate of linear electron transport. The double mutant *Δrh1/2* had the lowest Φ_PSII_ under control conditions, while this same parameter was the highest under salt stress condition. This discrepancy was caused by the fact that Φ_PSII_ decreased under salt stress conditions in all genotypes in comparison to control condition, while, in *Δrh1/2*, it was kept the same (Supplementary Fig. 5c).

The photochemical quenching (qP) indicates proportion of PSII reaction centres that are open and the lower values indicate increased excitation pressure on PSII. This was again found in the *Δrh2* mutant under control conditions, which is in accordance with decreased F_v_/F_m_ values. More oxidized PSII centres were found in the *Δrh1/2* mutant under high light conditions. The non-photochemical quenching parameter (NPQ) is related to heat dissipation in PSII. In *Δrh2* mutants, the values were lower under high light and salt stress conditions. Increased NPQ was found in the *Δrh1/2* double mutant under control and high light conditions, but during salt stress conditions, the parameter was the lowest (Supplementary Fig. S11).

In conclusion, *Δrh1* and WT genotypes do not differ in any photosynthetic parameters and seem to be very similar under different growth conditions, while the *Δrh2* and *Δrh1/2* mutants differed from WT and *Δrh1*, often exhibiting opposite trends relative to each other.

**Fig. 6:**
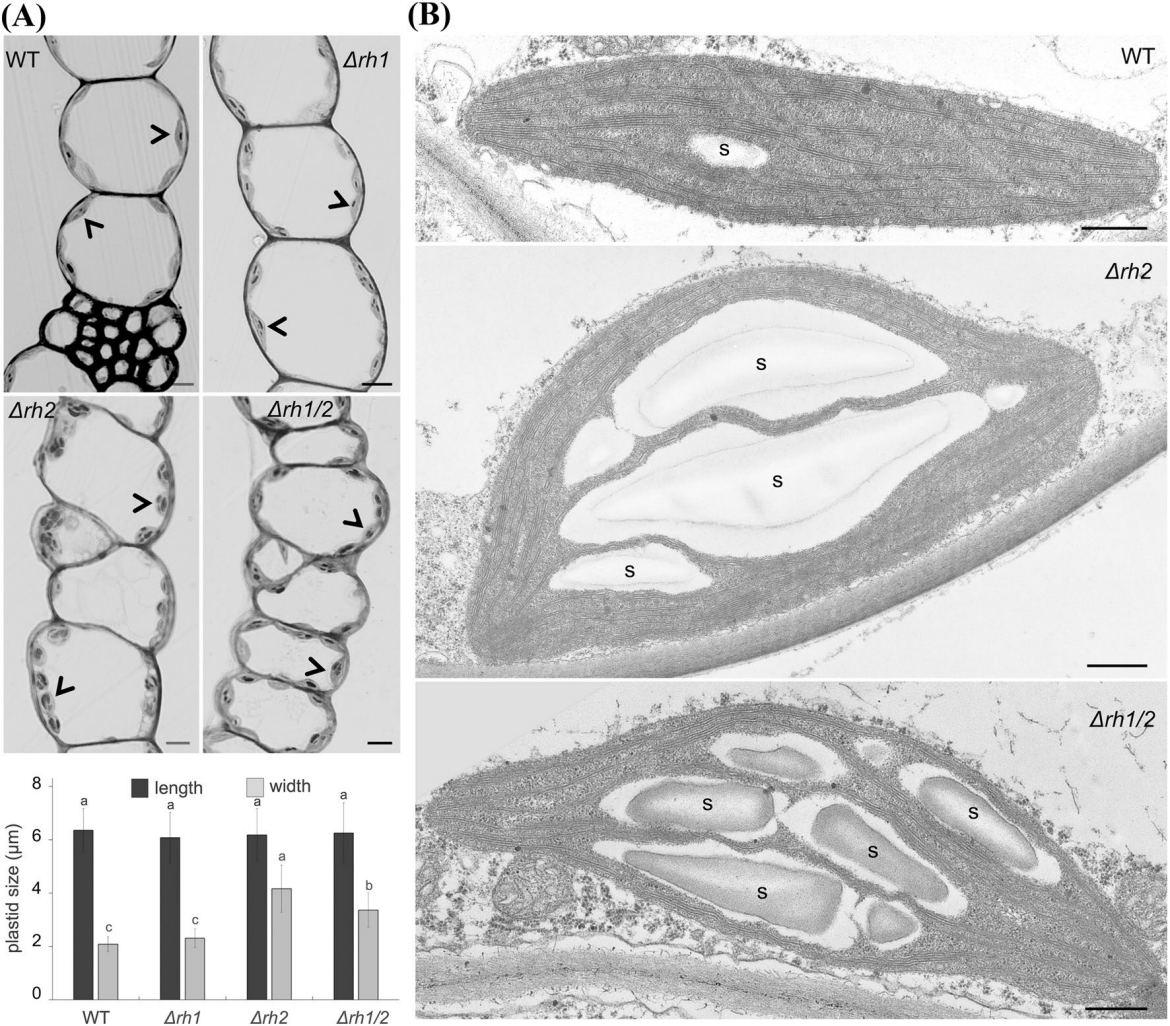
Chloroplast morphology. **a** Semithin sections of *Physcomitrium patens* phyllids from one month old gametophores grown under standard conditions. Plant genotypes are indicated in the individual panels. Each arrowhead points to an individual plastid. Starch grains appear as dense structures inside the plastids. Plastid size is represented by length and width values in the graph below. One hundred plastids from each genotype were measured. Different letters indicate significant differences at *P* < 0.05 within individual treatment (ANOVA, Tukey-test), (n = 100).** b** Representative transmision electron micrographs of plastids from WT, *Δrh2* and *Δrh1/2* phyllids. Starch grains are indicated by s. Scale bars: 5 μm in **a**, 500 nm in **b**

To further explore change in photosynthetic performance of the helicase mutants, we performed anatomical and ultrastructural observations of phyllid chloroplasts. A closer examination of plastid morphology in plants growing under standard conditions revealed morphological changes in both *Δrh2* and *Δrh1/2* mutants. Compared to WT and *Δrh1*, plastids in *Δrh2* and *Δrh1/2* are round-shaped with increased width and a visibly larger content of starch grains. (Fig. 6a). The ultrastructural observations of plastids further demonstrated the over-accumulation of starch in *Δrh2* and *Δrh1/2* mutants compared to WT. On the other hand, the plastid internal membrane system remained well organized in the mutants and we did not observe any abnormalities in plastid stroma (Fig. 6b).

### *P. patens Δrh2* and *Δrh1/2* display fertility and sporophyte development impairments

**Fig. 7:**
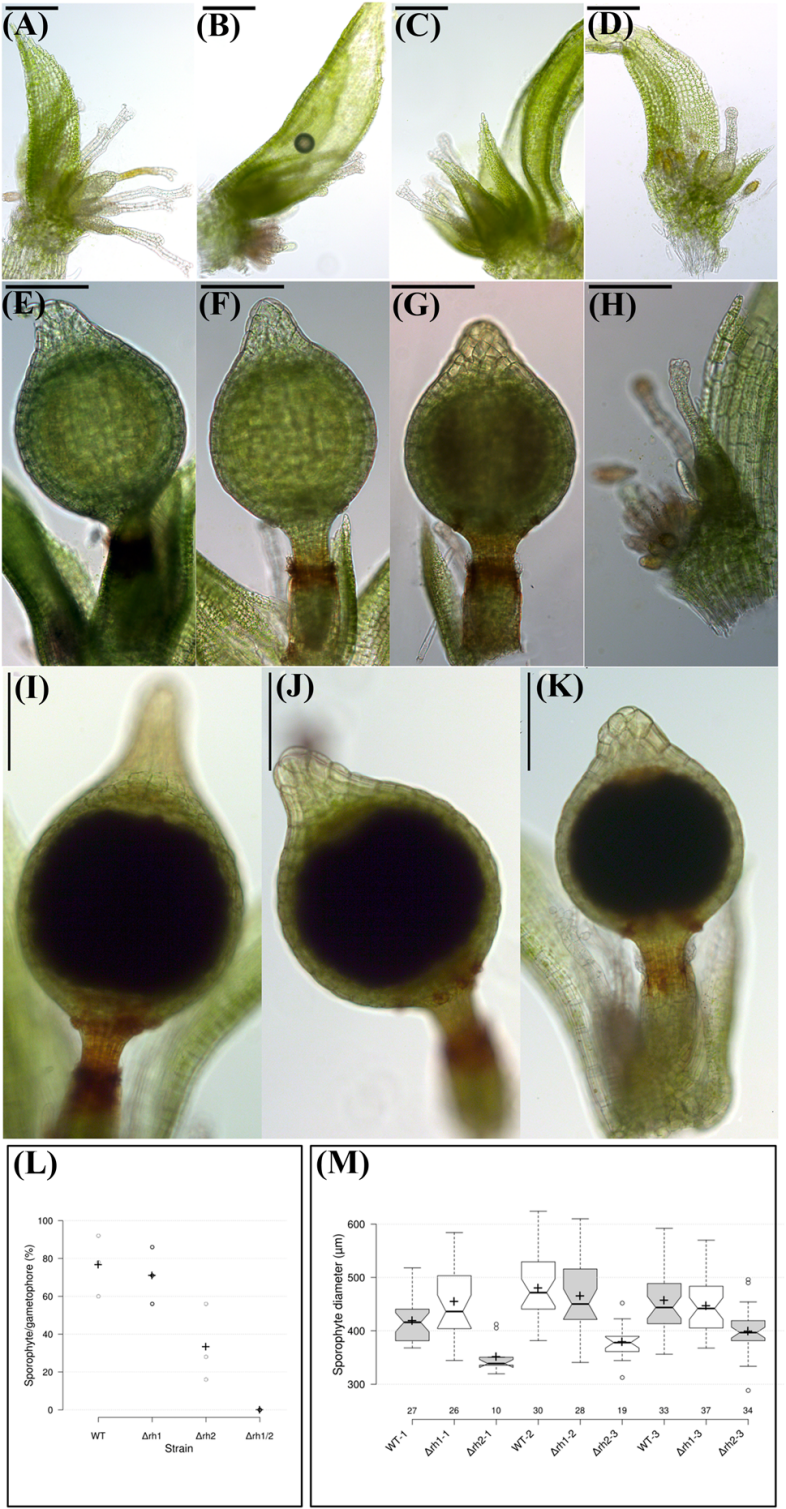
Sporophyte formation in WT, *Δrh1, Δrh2* and *Δrh1/2*. *Physcomitrium patens* gametophore apices of plants grown 35 days in LD at 22 °C followed by either 21 days in SD at 15 °C (**a–d**), 35 days in SD at 15 °C (**e–h**) or 61 days in SD at 15 °C (**g–i**). These pictures are representative of more than 600 gametophore apices grown in three independent sporulation experiments. After 21 days in cold conditions, gametangia are detectable in mutant plants in similar time frame in contrast to WT plants. **a** WT. **b** *Δrh1*. **c** *Δrh2*. **d** *Δrh1/2*. After 35 days in inducible conditions, green sporophytes are observable on WT, *Δrh1* and *Δrh2* apices, but not on *Δrh1/2*. **e** WT sporophyte. **f** *Δh1* sporophyte*.* **g** *Δrh2* sporophyte. **h** *Δrh1/2* apex with unfertilized archegonia. After 61 days in cold conditions, brown sporophytes were observed on WT, *Δrh1* and *Δrh2* apices. **i** Wild-type brown sporophyte. **j** *Δrh1* brown sporophyte*.* **k** *Δrh2* brown sporophyte*.* Bar in all pictures: 200 μm. **l** Fertility rate expressed in % of gametophores forming a sporophyte. Each dot represents an independent sporulation experiment average with n = 75–125. The black cross represents the average value of the triplicate. *T*-test indicates that *Δrh2* is significantly different from either WT or *Δrh1* (*p* < 10^−3^*)*. **m** Box plot diagram showing sporophyte diameter distribution (n indicated along the y axis) in three independent sporulation experiments (1–3). *T*-test results indicate that *Δrh2* dimensions are significantly different from either WT or *Δrh1* in three independent biological replica (*p* < 10^−3^)*.* The plot was generated using the online tool BoxPlotR: a web-tool for generation of box plots (http://shiny.chemgrid.org/boxplotr/)

To evaluate the impact helicase gene deletion on fertility and sporophyte development in Δr*h1*, Δr*h2* and *Δrh1/2*, we exposed them to the standard sexual reproduction procedure (Perroud et al. 2011). In three independent culture replications, WT, *Δrh1*, *Δrh2* and *Δrh1/2* produced similarly gametangia after 3 weeks in inductive conditions (Fig. 7a–d). After 5 weeks of inducing conditions, green sporophytes were observed in WT, *Δrh1* and *Δrh2* (Fig. 6e–g) as *Δrh1/2* did not display sporophyte (Fig. 7h). After nine or 14 of weeks in inductive conditions, *Δrh1/2* remained sporophyte-less, an indication that these two specific RNA helicases are either involved in sexual reproduction or/and sporophyte development. WT, *Δrh1* and *Δrh2* brown sporophyte (Fig. 7i–k) (Hiss et al. 2017) number and size were analyzed after 9 weeks in inductive conditions. *Δrh2* cultures diverged clearly from WT and *Δrh1*. *Δrh2* fertility, as measured by the number of sporophytes per gametophore, was reduced to 33% compared to 77% and 71%, respectively, in WT and *Δrh1* (Fig. 7l). Also, *Δrh2* sporophyte size, measured as diameter of the capsule, was consistently reduced by 10 to 20% across replica compared to either WT or *Δrh1* (Fig. 7m). The reduction in sporophyte number and size further implicates a functional role for RNA helicase in both sexual reproduction and sporophyte development in *P. patens*. We subsequently evaluated spore germination from selfed sporophyte of WT, *Δrh1* and *Δrh2*. The three different spore types germinated into normal looking sporelings (Supplementary Fig. 11a–c). However, germination rates differed. WT and *Δrh1* spore sets from multiple sporophytes displayed a mean germination rate of 80 and 82%, respectively, a value range in accordance with previous observations for WT spore germination after 1 week of growth (Engel 1968; Perroud et al. 2019). In contrast, the *Δrh2* mean spore germination rate was reduced to 49% and spore sets from different sporophytes displayed a highly variable germination rate ranging from 2 to 75% (Supplementary Fig. S12d). Morphologically, WT and *Δrh1* spores were undistinguishable in appearance (round to slightly ovoid with a regular surface sporopollein deposit) with a diameter of 31.25 ± 2.74 μm and 29.94 ± 3.04 μm, respectively (Supplementary Fig. S13, a–h). *Δrh2* spore size distribution presented a larger variation that appeared to be dependent on the specific sporophyte analyzed (Supplementary Fig. S13, g–p and q). Overall, *Δrh2* displayed a smaller average spore size with 28.1 ± 4.99 μm. Additionally, spore not only ranged from mature WT looking spores (Supplementary Fig. S13, i) to very small with a normal surface structure (Supplementary Fig. S13, j, m) but also displayed collapsed aspect with strong alteration of the spore surface (Supplementary Fig. S13, o, p).

To better understand the origin of *Δrh1/2* self-sterility, we conducted crossing experiments with either known fertile males with a visually tractable marker or a sterile male mutant. Using standard co-cultivating procedure and time-extended cultures, no sporophyte development was observed on *Δrh1/2* gametophores when co-cultivated with *Vx-red* (Perroud et al. 2011) or *Re-mCherry* (Perroud et al. 2019) as these two well-characterized fertile strains selfed normally in the co-cultures (sporophytes forming on 100% of gametophores with more than 15 phyllids). Hence *Δrh1/2* archegonia, albeit displaying a normal morphological development are apparently sterile. In parallel, *Δrh1/2* was co-cultivated with *ccdc39*, a male sterile mutant with fully functional archegonium (Meyberg et al. 2020). Similarly to selfing trials, Δr*h1*/*2* gametophores did not form any sporophyte and a single sporophyte was detected on *ccdc39*. Progeny analysis indicated that sporophyte was a very rare *ccdc39*-selfed sporophyte. These results indicate that *Δrh1/2* spermatozoids, however present, are infertile.

## Discussion

*Pp*RH1 and *Pp*RH2 proteins are members of the DEAD-box RNA helicases and contain a C-terminal GUCT domain. These proteins, together with their GUCT-containing orthologues, constitute a clearly separated and distinct major group that branches deeply in the phylogenetic tree of *Viridiplantae* DEAD-box RNA helicases (Fig. 1), suggesting a common and conserved function for these proteins. Using mass spectrometry analysis, GUCT was previously identified as a nucleolar protein domain in humans (Andersen et al. 2002). In the present study, we show that *Pp*RH1 localizes to the nucleus, as does the *A. thaliana* orthologue AtRH7 (Lorković et al. 1997), providing experimental validation for the predicted localization of GUCT-containing DEAD box RNA helicase to this organelle. GUCT domains appear to be present only in proteins containing domains with annotations related to RNA biology and mainly restricted to eukaryotic proteins, the latter also supported by a previous in silico analysis (Staub et al. 2004). Information regarding the function of the GUCT domain is currently absent from the scientific literature. The GUCT domain of the human ATP-Dependent RNA Helicase DDX50 (RH-II/Guβ) protein adopts the RNA recognition (RRM) fold (Ohnishi et al. 2009), however, the critical amino acids, documented to be necessary for binding and coordinating RNA, are absent, and it has been suggested that the domain, in DDX50, might rather function in general protein binding. Functional studies of human DDX50 in HeLa cells have shown that the protein is necessary for cell growth and cell cycle progression (Ou et al. 1999) and that the protein is involved in ribosomal RNA production (Henning et al. 2003). Similarly, the AtRH7 protein, predominantly expressed in cells and in regions of cells undergoing active cell division, is also involved in ribosomal RNA production in *A. thaliana* (Huang et al. 2016). Interestingly, our analysis shows that *Pp*RH1 accumulates to a higher level in the cells of the growing protonemal filaments (Fig. 3). Combined, these results suggest that the function of GUCT-containing RNA helicases is associated with cells actively expanding in mosses.

The functional characterization of plant DEAD-box RNA helicases belonging to sub-clade I is still limited to *A. thaliana* (Lorković et al. 1997; Kim et al. 2013; Huang et al. 2016; Liu et al. 2016) and *P. patens* (this work). In addition, *PpRH1* and *PpRH2* represent paralogues in the moss in contrast to the single orthologues identified in *A. thaliana*, *O. sativa*, *M. polymorpha* and *M. viride* (Fig. 1, Supplementary Fig. S1), which raises questions about their functional conservation. When it comes to biological outcome of their molecular function (r-RNA processing and hence ribosome assembly), diverse developmental processes appear affected by the *A. thaliana* ortholog *AtRH7*. *atrh7* mutants display reduced fertility, shorter siliques with shrunken seeds and variable defects in embryo patterning (Huang et al. 2016; Liu et al. 2016). It has been proposed that a developmental role of AtRH7 might be associated with auxin signaling via translational control of auxin response factors (ARFs) (Huang et al. 2016). This hypothesis was based on observations where ribosome protein mutants that exhibit reduced ARF content, and hence altered auxin response, show similar phenotypes as *atrh7* mutants (Rosado et al. 2012; Huang et al. 2016). In *P. patens*, the absence of *PpRH1* had no detectable effect on growth and development throughout the moss life cycle. This is in sharp contrast with diverse phenotypic consequences of the loss of *PpRH2*, or both genes in the double mutant, which indicates intricate functional interconnection. While, in *Δrh2*, the protonemal filaments show accelerated side-branch growth with actively expanding caulonemal cells, the protonematal growth and caulonemal development is strongly reduced in the *Δrh1/2* double mutant. Although auxin signaling has been attributed to control of protonemata growth and development in numerous studies (reviewed by Thelander et al. 2018), a link between auxin and ribosomal RNA processing in representatives of early diverging land plants is still missing. Similarly, as in protonemata, deletion of *PpRH2* alone*,* and both *PpRH1* and *PpRH2* in the double mutant, caused opposite rather than cumulative effects on gametophore growth (Figs. 4, S6). One common feature in *Δrh2* and *Δrh1/2* mutants was altered plastid morphology and increased accumulation of starch (Fig. 6). Impaired starch degradation has been recently reported in *P. patens glucan, water dikinase* (*Ppgwd*) mutants (Mdodana et al. 2019), however, in that case, gametophore formation was almost completely blocked. Despite dramatic developmental differences between *Δrh1, Δrh2* and *Δrh1/2* mutants, their photosynthetic performance is rather similar, which indicates that chloroplast biogenesis is not a primary cause of the mutant phenotypes.

In *A. thaliana*, the *atrh7* mutant exhibits considerably increased developmental abnormalities after prolonged growth at low temperature (Huang et al. 2016; Liu et al. 2016). This effect was associated with a cold-induced role of AtRH7 in rRNA processing. In *P. patens*, 24 h cold treatment had no considerable effect on *PpRH1* and *PpRH2* transcript levels and long-term cultivation at 10 °C did not change phenotypical characteristics of the mutants gametophores. In addition, the *PpRH1* and *PpRH2* deletion mutants showed a similar growth response as WT when cultivated under low-light, high-light or osmotic stress conditions. This indicates that the role of *Pp*RH1 and *Pp*RH2 is associated with default developmental control rather than stress-induced protective mechanisms. Hypothetically, cold responsiveness function has been acquired during the evolution of *A. thaliana* ortholog *AtRH7* as a novel element in addition to it’s developmental role. The DEAD-box RNA helicase elF4A protein, which consists of the helicase core only, was recently reported to be involved in stress management and cell cycle progression in *P. patens* (Tyagi et al. 2020). The difference in *Pp*RH1/*Pp*RH2 and elF4A response to stress suggests functional specialization of these two groups of DEAD-box RNA helicases in *P. patens* and is also supported by the phylogenetic analysis that clearly sorts the *Pp*RH1/*Pp*RH2 and elF4A proteins into distinct clades (Supplemetary Fig. S1).

Finally, *Pp*RH1 and *Pp*RH2 genetic analysis revealed an intriguing functional pattern The two paralogs *Pp*RH1 and *Pp*RH2 are 82% identical and possess an identical domain topology. But, their specific deletion generates two different phenotypes. *Δrh1* does not display any morphological detectable divergence to WT. In contrast, *Δrh2* presents developmental differences with WT in virtually all gametophytic and sporophytic tissues of *P. patens*. Additionally, *Δrh1/2* showed even more severe growth defects than *Δrh2* and was completely sterile. This implies that, despite being nonessential for normal plant development, *Pp*RH1 possesses a partial functionality in the *Δrh2* background. Two potential hypotheses could explain these observations. Perhaps the accumulated amino acid differences between the two paralogues reflect the beginning of neo-functionalization, in which *Pp*RH1 has acquired a novel function not detected by our analysis and is already sufficiently diverged to only partially complement *Pp*RH2 absence. Alternatively, *Pp*RH1 is in an early phase of decay that will ultimately lead to the formation of a true pseudogene. This second hypothesis is further supported by the differential expression patterns of these two genes. *PpRH1* transcripts accumulate to higher levels than those of *PpRH2* in all investigated tissues. This could represent a loss of *PpRH1* regulation either through transcript stability or promoter regulation, a step in the process of gene degeneration toward the formation a fully inactive pseudogene.

## Material and method

### Bioinformatic analysis

RNA helicases from *C. reinhardtii* (v.5.5), *M. polymorpha* (v3.1), *P. patens* (v3.3), *O. sativa* (*v7_JGI)*, *A. thaliana* (TAIR10) and *S. lycopersicum* (iTAG2.4) were identified and downloaded from the Phytozome12 database using Pp3c20_20710 (*Pp*RH1) as query in pBLAST searches. *M. viride* RNA helicase sequences were downloaded from the MVGE (v1.0) database at http://www.elabcaas.cn/alga/index.html (Liang et al. 2020) using the same approach. Each of the sequences was submitted to Pfam (v.33.1) to identify and annotate domain modules and only domains with significant annotations and containing the DEAD signature sequence in motif II (Fig. 1a) in the helicase core were further selected (Supplementary Data S1). The helicase core (e.g. the subsequence spanning from 10 amino acids upstream to 10 amino acid downstream of the DEAD and helicase_C domains, respectively) was further extracted and aligned using MAFFT (v.7) and the E-Ins-I algorithm (Katoh and Standley 2013). Reconstruction of maximum likelihood (ML) phylogeny of the helicase core was performed using neighbor joining and the WAG substitution model in the CLC genomic Workbench package with 1000 bootstrap iterations. Subcellular localization signals were predicted using the Plant-mPLoc server (Chou and Shen 2010) at http://www.csbio.sjtu.edu.cn/bioinf/plant-multi/.

### Plant material and plant culture

*Physcomitrium patens* (previously known as *Physcomitrella patens*) (Medina et al. 2019) Gransden wild-type (WT), pedigree Gd_JP_St Louis (Haas et al. 2020) was used as starting plant material for this study. Maintenance and amplification tissue culture for the WT and mutant strains were performed either directly on BCDA medium (Cove et al. 2009) for spot inoculum or on cellophane overlaying the medium for blended tissue used for protoplasting. Standard culture conditions are long-day light (16 h light / 8 h darkness) with a light intensity of 70 μmol s^−1^ m^−1^ at a constant temperature of 24 °C. All vegetative tissue phenotypic analyses were performed on BCD medium (Cove et al. 2009). Plants harvested for RT-qPCR analyses were grown under standard conditions unless otherwise specified. For long-term stress treatments, spotted cultures were cultivated for 2 weeks on BCD media under long-day light with intensities 5 and 140 μmol s^−1^ m^−1^ at 24 °C and on media supplemented with 90 mM NaCl, respectively. For short-term stress treatments, blended protonemata were first grown on BCD media overlaid with cellophane for 2 weeks under standard conditions. Then, the cultures were transferred to fresh BCD media and media supplemented with 10 μM abscisic acid (Sigma-Aldrich). Part of the cultures was transferred to a 10 °C bath. Plants were thusly treated for 24 h before harvesting for further analyses.

*Physcomitrium patens* culturing, both WT and mutants, was performed to access fertility as previously described (Perroud et al. 2011, 2019). Shortly, after 5 weeks of growth in standard conditions, single or double mutant strain cultures were transferred to gametangia inductive condition (Hohe et al. 2002), at 15 °C with a short day light cycle of 8 h light/16 h dark regime with a reduced fluence rate of 20 µmol m^−2^ s^−1^ white light, for the rest of the experiment. Two weeks later, plants were watered with sterile water and put back in inductive conditions. The presence of gametangia was evaluated after 3 weeks in inductive conditions and the standard green sporophyte observation time point was performed 2 weeks later (i.e. after 5 weeks). Brown sporophytes were counted on gametophores displaying more than 15 phyllids, the percentage of sporophyte-bearing gametophores was used as proxy for fertility. Sporophyte diameter was measured at the middle of the sporogenic domain of the sporophyte, perpendicular to its axis of development. For spore germination assay, sporophytes were harvested under sterile conditions and were stored for at least a week in the dark at 4 °C before evaluation of spore germination (Perroud et al. 2019).

### Plasmid vector constructions

Plasmid vectors were constructed using recombination-based In-Fusion® Cloning technology (Takara Bio Europe, SAS, France) according to the manufacturer´s instructions. The sequences of the oligonucleotide primers used in vector constructions are listed in Supplementary Table S3. Final plasmid constructs were verified by restriction digest analysis and sequencing.

The two-knockout (KO) vectors pBHRF_hel1_KO and pBNRF_hel2_KO (Supplementary Fig. S14) were generated to delete the *Pp3c20_20710* and *Pp3c10_20840* genes, respectively. To generate the vector pBHRF_hel1_KO, the 5′ and 3′ targeting sequences (TGS) were PCR-amplified with genomic DNA extracted from WT *P. patens* Gransden using primer pairs SP_5′_ARH1 and ASP_5′_ARH1, and SP_3′_ARH1 and ASP_3′_ARH1, respectively. The PCR-amplified 5′ and 3′ TGSs were cloned sequentially into *NruI*- and *SpeI*-linearized plasmid pBHRF (Schaefer et al. 2010), respectively, resulting in vector pBHRF_hel1_KO. To generate the vector for pBNRF_hel2_KO, the 5′ and 3′ TGS were PCR amplified using primer pairs SP_5′_ARH2 and ASP_5′_ARH2, and SP_3′_ARH2 and ASP_3′_ARH2, respectively. The PCR-amplified 5′ and 3′ TGSs were inserted sequentially into plasmid pBNRF (Schaefer et al. 2010) linearized by inverse PCR using primer sets pBHRF-5In-SP and pBHRF-5In-ASP, and pBHRF-1-SP and pBHRF-1-ASP to clone the 5′ and 3′ TGS fragments, respectively, resulting in KO vector pBNRF_hel2_KO.

To identify the subcellular localization of the *Pp*RH1 protein, we generated the knock-in vector, phel1_GFP_KI (Supplementary Fig. S14), designed to insert the coding sequence of a trimeric enhanced green fluorescent protein (3XmGFP) in-frame with the 3′ end of *PpRH1*. Additionally, the fluorescent tag was separated from the native protein by a linker sequence corresponding to amino acids Gly-Gly-Gly-Gly-Ser. Prior to generating the final phel1_GFP_KI vector, the 3xmGFP fragment was PCR amplified from vector p3XmEGFP-L5L4 (generous gift from M. Bezanilla and L. Vidali) using primers Ppf1 and Ppf2 and then cloned into vector pBHRF (Schaefer et al. 2010) using *NruI* and *XhoI* to generate vector p3xGFP-LoxP_Hygro_LoxP. The phel1_GFP_KI vector was then constructed in the following way: the 5′ TGS was first PCR amplified from gDNA extracted from WT *P. patens* Gransden using primer pair SP_5′-KI and ASP_5′-KI. The reverse primer (ASP_5′-KI) was designed to harbour a 5′ extension to add the 15-bp linker sequence to the 3′ end of the amplified product. The resulting PCR fragment (1348 bp) was sub-cloned into the Zero Blunt PCR vector (Invitrogen) generating plasmid pPCR_5′TGSLinker. Finally, the 5′ TGS fragment was PCR amplified with pPCR_5′TGSLinker DNA as template using primer pairs SP_5′Inf-KI and ASP_5′Inf-KI containing 5′ 15-bp extensions to allow In-Fusion cloning into vector p3xGFP-LoxP_Hygro_LoxP vector. Similarly, primer pair SP_3′Inf-KI and ASP_3′Inf-KI were used to PCR amplify the 3′ TGS using *P. patens* WT gDNA as the template. The resulting 5′ and 3′ TGSs fragments were In-Fusion cloned sequentially into p3xGFP-LoxP_Hygro_LoxP, linearized with *NruI* and *SpeI*, respectively, leading to the final vector phel1_GFP_KI.

### Plant transformation

*Physcomitrium patens* protoplast production and transformation was performed according to Schaefer and Zrÿd (Schaefer and Zrÿd 1997) as modified by Cove and collaborators (Cove et al. 2009). Plasmids pBHRF_hel1_KO, pBNRF_hel2_KO and phel1_GFP_KI were linearized with restriction enzyme pairs *BmrI*/*PacI*, *SmaI*/*PacI* and *HpaI*/*PmlI*, respectively (Supplementary Fig. S14). The double mutant (*Δrh1/2)* was generated by transforming pBHRF_hel1_KO into the *Δrh2/cre* background (Supplementary Fig. S5). Fifteen μg of linearized plasmid DNA was used per transformation. Briefly, transformed protoplast regeneration and selection was performed by transferring the tissue to different media according to the following sequence: 7 days of protoplast regeneration on protoplast regeneration medium for the bottom layer (PRMB), 7 days of selection on BCDA medium supplemented with the appropriate antibiotic, 14 days of growth on BCDA medium, and 7 days on BCDA medium supplemented with the appropriate antibiotic. Resistant plants were individually transferred to fresh BCDA medium and used for genotyping after sufficient growth.

The Cre recombinase procedure to remove the resistant marker from the primary transformant was performed as described previously (Trouiller et al. 2006) with minor modifications. Transformed tissue was grown as the WT, and protoplast production and transformation was performed using 20 μg of the plasmid pAct-Cre (Trouiller et al. 2006). Diluted protoplast suspensions were plated on cellophane-coated medium (approximately 25,000 counted protoplasts per 9-cm Petri dish) to avoid mixing of regenerated plants. Protoplast regeneration and the test procedure were performed as follows: (1) 4 days of protoplast regeneration on PRMB medium; (2) 4 days of protoplast growth on BCDA medium; (3) individual plants transferred to fresh BCDA medium and growth for 8 days and (4) replica plating of each individual plant onto BCDA medium and BCDA medium supplemented with the appropriate antibiotic. Strains showing loss of antibiotic resistance were selected and grown until sufficient tissue was available for PCR genotyping.

### Molecular characterization of the *P. patens* mutants

All mutants were subjected to three rounds of PCR genotyping (Supplementary Fig. S3), using the Phire Plant Direct PCR kit (Thermo Fisher Scientific, Schwerte, Germany) according to the manufacturer’s instructions. The primer sequences used for genotyping are provided in Supplementary Table S3.

The *Δrh1*, *Δrh2* and *Δrh1/2* mutants were first genotyped for loss of locus using primers specific to the deleted gene (for Pp3c20_20710, primers F-KO1_GS and R-KO1_GS; for Pp3c10_20840, primers F-KO2_GS and R-KO2_GS). All plants displaying loss of the WT locus were further genotyped for 5′ and 3′ targeting events. 5′ and 3′ targeting for putative Pp3c20_20710 deletion mutants (*Δrh1*) were performed using primers setts F-KO1_GT and 35S-R, and Term-F and R-KO1_GT, respectively. For putative Pp3c10_20840 deletion mutants (*Δrh2*), primer sets F-KO2_GT and 35S-R and Term-F and R-KO2_GT were used for 5′ and 3′ targeting, respectively. Plants positive for both 5′ and 3′ targeting were finally genotyped to identify single-copy insertions at the Pp3c20_20710 and Pp3c10_20840 loci using primers F-KO1_GT and R-KO1_GT and F-KO2_GT and R-KO2_GT, respectively. Genotyping for putative double mutants (*Δrh1/2)* was performed as described previously for the single Pp3c20_20710 deletion mutant (*Δrh1)*. Before transformation of *Δrh2/cre* to create *Δrh1/2*, the *Δrh2*/cre mutant was verified by Southern Blot hybridization. Genomic DNA was extracted using the Nucleon PhytoPure Genomic DNA Extraction Kit (GE Healthcare). Southern hybridization was performed as described by Perroud and Quatrano (2006) using 1 µg of XhoI-digested DNA. Two hybridization probes corresponding to the 5′ and 3′ targeting sequences were labelled with digoxigenin using the DIG Probe PCR synthesis kit (Roche) according to the manufacturer’s instructions. The construct pBNRF_hel2_KO was used as template for PCR amplification of the probes. The sequences of the primers used to generate the probes (5′TGSp-F/5′TGSp-R and 3′TGSp-F/3′TGSp-R primer sets for the 5′TGS and 3′TGS probes, respectively) are provided in Supplementary Table S3, and the hybridization sites of the probes are shown schematically in Supplementary Fig. S5.

For putative *rh1-3xgfp* mutants, first genotyping was performed to verify insertion of *3XmEGFP* using primer pair F-KI_GS and R-KI_GS (Supplementary Fig. S3). Mutant lines showing the expected amplicon size were subjected to a second round of PCR genotyping to verify 5′ and 3′ targeting events using primer pairs F-KI_GT and 35S-R, and Term-F and R-KI_GT, respectively. In the third round of PCR genotyping, primer pairs F-KI_GT and R-KI_GT were used to confirm single copy insertion at the locus.

### Reverse-transcriptase quantitative PCR (RT-qPCR)

Plant material was frozen in liquid nitrogen and stored at -70 °C prior to RNA extraction. Total RNA was extracted in three independent biological replicates using either the Plant RNeasy kit (Qiagen) or the Spectrum Plant Total RNA kit (Sigma–Aldrich) according to the manufacturer’s instructions. After analysis of RNA purity and integrity 500 or 1000 ng DNaseI-treated RNA was reverse transcribed using either 100 units of SuperScript™ III reverse transcriptase (Thermo Fisher Scientific) primed with 50 μM random hexamers primers at 55 °C for 60 min or using GoScript™ Reverse Transcription System (Promega) according to the manufacturer’s protocol. Transcript level analysis was performed in triplicate using the HOT FIREPol® EvaGreen® qPCR Mix Plus (ROX) system (Solis Biodyne) on the 7500 Fast Real-Time PCR Systems (ABI) using the following thermal profile for all targets: 95 °C for 15 min followed by 40 cycles of 95 °C for 30 s, 62 °C for 20 s, and 72 °C for 32 s. For one set of experiments, qPCR analysis was performed using the Maxima SYBR Green/ROX qPCR Master Mix system (Thermo Fisher Scientific) at an annealing temperature of 60 °C. Final post-PCR dissociation analysis was included for each run. Mean PCR efficiency values (E) for each targets were determined using the LinRegPCR software v11.0 (Ruijter et al. 2009) and by dilution curves prior to qPCR analysis. Relative transcript levels of targets were normalized to the transcript level of RIBOSOMAL PROTEIN S9 (RSB; Pp3c18_1350V3.1) (Xiao et al. 2011). Fold change expression values were calculated relative to the WT, which was set to 1, using the Pfaffl method (Pfaffl 2001). The Student’s *t* test was used for statistical analysis (Statgraphics centurion 15.2.05). cDNA targets were amplified using the following gene-specific primer pairs: for *PpRH1*, Arh1q-F and Arh1q-R; for *PpRH2*, Arh2q-F and Arh2q-R; and for *PpRSB*, RSBq-F and RSBq-R (Supplementary Table S3).

### Chlorophyll fluorescence measurements

The moss (*P. patens*) was dark-adapted for 40 min. The Petri dishes were open prior measurements. The chlorophyll *a* fluorescence was measured using fluorocamera FluorCam 800 MF (Photon System Instruments, Drášov, Czech Republic). Minimal fluorescence (F_0_) was measured at 0.25 µmol m^−2^ s^−1^ PAR and λ = 620 nm. Then, maximum fluorescence (F_m_) was measured by a saturation pulse at 3200 μmol m^−2^ s^−1^ PAR, 800 ms duration and λ = 450 nm, and the maximum quantum yield (variable/maximum fluorescence, F_v_/F_m_) was calculated as (F_m_ − F_0_)/F_m_. For analysis of the quenching mechanism, actinic light was turned on for 10 min (70 μmol m^−2^ s^−1^ PAR, λ = 620 nm) and ten saturation pulses were triggered to estimate maximum chlorophyll fluorescence in the light-adapted state (${\text{F}}_{{\text{m}}}^{\prime }$, 3200 μmol m^−2^ s^−1^ PAR, 800 ms duration, λ = 450 nm). The actinic light was then turned off and F_0_′ was measured. The effective photochemical quantum yield of photosystem II (Φ_PSII_), photochemical quenching (qP) and non-photochemical quenching (NPQ) were calculated according to Maxwell and Johnson (2000).

### Standard microscopy procedure

Routine tissue check and early phenotype analyses were performed with a Leica S8 APO binocular (Leica, Wetzlar, Germany). Microscopic picture were taken with an upright DM6000 equipped with a DFC295 camera (Leica, Wetzlar, Germany). The Leica Application Suite version 4.4 was used as acquisition tool. Size measurement was performed using the measure tool from Image J1.52t (Rasband 2018) on unmodified primary pictures. Subsequent image processing (brightness and contrast adjustment) was performed with Adobe Photoshop Suite.

### Plastid morphology and ultrastructure observation

For microscopy studies, whole cut gametophores were fixed with 3% (w/v) glutaraldehyde and 1% fresh prepared paraformaldehyde in 100 mM MTSB buffer (Vitha et al. 2000) for three hours and after washing postfixed with 1% (v/v) osmium tetroxide in the same buffer for two hours. After dehydration in ethanol, samples were embedded in Spur’s resin (Spurr 1969). Semithin (1 μm thick) and ultrathin sections were cut by LKB 8800 Ultratome III. Semithin sections were stained with 1% toluidine blue 0 in 1% sodium borate and poststained with 0.1% aqueous basic fuchsin and examined in an Olympus BX61 epifluorescence microscope with an Olympus UPlanSApo 60X/1.35 Oil objective lens and under bright light illumination. Images were acquired with a Levenhuk M 1400 Plus digital camera and a LevenhukLite software. The size of plastids was analyzed on semi-thin sections using ImageJ software. Ultra-thin Sects. (50 nm thick) were contrasted with 2% uranyl acetate dissolved in 50% ethanol and with lead citrate (Reynolds 1963) for 20 min each and studied in a Jeol 100 CX electron microscope at 100 kV.

### Confocal microscopy procedure

Confocal microscopy acquisitions were performed using a Leica TCS SP8X as described (Perroud et al. 2020) with fluorescence excitation and signal acquisition wavelength modifications. For live imaging of 3× GFP tagged protein, excitation wavelength was set to 489 nm and the signal acquisition was performed between 510 and 550 nm using the HyD™ detector. The acquisition signal was gated between 0.5 and 6 ns after the laser pulse. Chloroplast auto-fluorescence was acquired in parallel using the same excitation wavelength with an acquisition window between 660 and 710 nm using a standard PMT. For all images scan speed was set 400 Hz (400 lines/s) at a resolution at 1024 × 1024 pixels and four lines averaging per single image was used. Post acquisition processing was performed using Image J1.52t (Rasband 2018) and assembled in PowerPoint 2016.

## Supplementary Information

Below is the link to the electronic supplementary material.

Supplementary file1 (PDF 38848 kb)
