# Supplementary material for: The nuclear GUCT domain-containing DEAD-box RNA helicases govern gametophytic and sporophytic development in Physcomitrium patens
Source: Plant Mol Biol. 2021 Apr 22;107(4-5):307–25. doi: 10.1007/s11103-021-01152-w (PMC8648619; doi:10.1007/s11103-021-01152-w)
Supplement: Supplementary file 1 — Supplementary file1 (PDF 38848 kb) [file 11103_2021_1152_MOESM1_ESM.pdf]

Supplementary Material

Article title: **The nuclear GUCT domain-containing DEAD-box RNA helicases govern gametophytic and sporophytic development in *Physcomitrium patens*.**

Authors: Pierre-François Perroud[1,a], Viktor Demko[2,3], Ako Eugene Ako[4,b], Rajendra Khanal[4,c], Boris Bokor[5], Andrej Pavlovič[6], Ján Jásik[3] and Wenche Johansen[4*]

The following supporting information is available for this article:

**Table S1**	Comparison of *PpRH1*and *PpRH2* exon structure
**Table S2**	List of DEAD-box RNA helicases used in the present study
**Table S3**	List of primers used in the study and their sequences
**Result S1**	Establishment and molecular characterizations of the *P. patens Δrh1*, *Δrh2*, *Δrh1/2* and *rh1-3xgfp* mutants
**Fig S1**	Phylogenetic tree of the DEAD-box RNA helicases core domain
**Fig S2**	*PpRH1* and *PpRH2* expression profiles in *P. patens* tissues as presented by the eFP browser
**Fig S3**	Targeted insertion and PCR genotyping of the *P. patens rh1-3xgfp* mutant line
**Fig S4**	Comparison of the GFP fluorescent signal between *rh1-3xgfp* and wildtype protonema
**Fig S5**	Targeted insertion and molecular characterization of the *P. patens Δrh1*, *Δrh2*, *Δrh1/2* and *Δrh2/cre* mutant lines
**Fig S6**	Long-term effect of reduced light on WT compared to *PpRH1* and *PpRH2* deletion mutant gametophores
**Fig S7**	Vegetative growth under low light
**Fig S8**	Vegetative growth under high light
**Fig S9**	Vegetative growth on 90 mM NaCl containing medium
**Fig S10**	Quantitative PCR analysis of *PpRH1* and *PpRH2* after ABA and cold treatment
**Fig S11**	Chlorophyll a fluorescence imaging and quantification
**Fig S12**	*Δrh2* spores display a reduced germination rate compared to WT and *Δrh1*

Fig S13 Mature *Δrh2* spores display an heterogeneous shape and size compared to WT and *Δrh1* spores

Fig S14 Cartoon of the plasmid constructs used to generate the *P. patens Δrh1*, *Δrh2*, *Δrh1/2* and *rh1-3xgfp* mutant lines

Supplementary Table S1

Comparison of *PpRH1* and *PpRH2* exon structure

	PpRH1	***PpRH2***
**Gene size**	3989 bp	4150 bp
**Genetic element**	**bp**	
Exon 1	492	492
Exon 2	129	129
Exon 3	141	141
Exon 4	112	112
Exon 5	71	71
Exon 6	375	375
Exon 7	759	768
Intron 1	262	382
Intron 2	134	150
Intron 3	124	124
Intron 4	130	128
Intron 5	223	188
Intron 6	124	105
5'UTR	392	444
3'UTR	521	541

Data based on annotations provide in Phytozome Release v.12.1 (*Physcomitrium patens* v3.3). *PpRH1* (Pp3c20_20710); *PpRH2* (Pp3c10_20840).

Table S2 List of DEAD-box RNA helicases used in the present study

	Gene ID	Domain structure	Size (aa)
ARABIDOPSIS THALIANA	AT4G16630	DEAD-helicase_C	789
	AT1G71370	DEAD-helicase_C-DUF4217	558
	AT3G22310	DEAD-helicase_C	610
	**AT5G26742**	DEAD-helicase_C-GUCT-Zf_CCHC	747
	AT5G63120	DEAD-helicase_C	482
	AT2G42520	DEAD-helicase_C	633
	AT2G01440	DEAD-helicase_C	973
	AT5G60990	DEAD-helicase_C	456
	AT3G22330	DEAD-helicase_C	616
	AT5G65900	DEAD-helicase_C-DUF4217	633
	AT3G18600	DEAD-helicase_C-DUF4217	568
	AT1G16280	DEAD-helicase_C	491
	AT3G16840	DEAD-helicase_C	827
	AT3G01540	WW-DEAD-helicase_C	618
	AT2G40700	DEAD-helicase_C-DUF4217	610
	AT2G33730	DEAD-helicase_C	733
	AT1G55150	DEAD-helicase_C	501
	AT5G14610	WW-DEAD-helicase_C	645
	AT1G77030	DEAD-helicase_C-DBP10CT	845
	AT5G08620	DEAD-helicase_C	563
	AT3G06480	WW-DEAD-helicase_C	1088
	AT3G58570	DEAD-helicase_C	646
	AT3G58510	DEAD-helicase_C	612
	AT4G09730	DEAD-helicase_C	621
	AT1G28180	DEAD-helicase_C	368
	AT1G72730	DEAD-helicase_C	414
	AT4G00660	DEAD-helicase_C	505
	AT5G05450	DEAD-helicase_C-DUF4217	593
	AT3G13920	DEAD-helicase_C	407
	AT5G63630	DEAD-helicase_C	522
	AT3G61240	DEAD-helicase_C	498
	AT5G08610	DEAD-helicase_C	850
	AT1G63250	DEAD-helicase_C	798
	AT1G31970	DEAD-helicase_C	537
	AT2G47330	DEAD-helicase_C	760
	AT1G20920	DEAD-helicase_C	828
	AT5G54910	DEAD-helicase_C-DUF4217	739
	AT5G51280	DEAD-helicase_C	1166
	AT2G07750	DEAD-helicase_C	845

	AT2G45810	DEAD-helicase_C	528
	AT1G54270	DEAD-helicase_C	407
	AT4G33370	DEAD-helicase_C	542
	AT3G09620	DEAD-helicase_C	990
	AT1G71280	DEAD-helicase_C-DUF4217	465
	AT4G34910	DEAD-helicase_C	626
	AT3G53110	DEAD-helicase_C	496
	AT1G59990	DEAD-helicase_C	567
	**AT5G62190**	DEAD-helicase_C-GUCT	671
SOLANUM LYCOPERSICUM	SlDEAD1	DEAD-helicase_C	605
	SlDEAD2	DEAD-helicase_C	1222
	SlDEAD3	DEAD-helicase_C	775
	SlDEAD4	DEAD-helicase_C	499
	SlDEAD5	DEAD-helicase_C	871
	SlDEAD6	DEAD-helicase_C	663
	SlDEAD7	DEAD-helicase_C	586
	SlDEAD8	DEAD-helicase_C-DUF4217	595
	SlDEAD9	DEAD-helicase_C	654
	SlDEAD10	WW-DEAD-helicase_C	707
	SlDEAD11	DEAD-helicase_C	613
	SlDEAD12	DEAD-helicase_C	652
	SlDEAD13	DEAD-helicase_C-DUF4217	567
	SlDEAD14	DEAD-helicase_C-Zf_CCHC	596
	SlDEAD15	DEAD-helicase_C	745
	SlDEAD16	DEAD-helicase_C	500
	SlDEAD17	DEAD-helicase_C	560
	SlDEAD18	DEAD-helicase_C	538
	SlDEAD19	DEAD-helicase_C	414
	SlDEAD20	DEAD-helicase_C-Zf_CCHC	596
	SlDEAD21	DEAD-helicase_C	414
	SlDEAD22	DEAD-helicase_C-DUF4217	598
	SlDEAD23	DEAD-helicase_C	631
	SlDEAD24	DEAD-helicase_C-DUF4217	755
	**SlDEAD25**	DEAD-helicase_C-GUCT-Zf_CCHC	747
	SlDEAD26	DEAD-helicase_C	414
	SlDEAD27	DEAD-helicase_C	554
	SlDEAD28	DEAD-helicase_C	559
	SlDEAD29	DEAD-helicase_C	800
	SlDEAD30	DEAD-helicase_C	489
	SlDEAD31	DEAD-helicase_C	440
	SlDEAD32	DEAD-helicase_C-DBP10CT	786
	SlDEAD33	DEAD-helicase_C	492

	SlDEAD34	DEAD-helicase_C	509
	SlDEAD35	DEAD-helicase_C	644
	SlDEAD36	DEAD-helicase_C	613
	SlDEAD37	DEAD-helicase_C	480
	SlDEAD38	DEAD-helicase_C	806
	SlDEAD39	DEAD-helicase_C	637
	SlDEAD40	DEAD-helicase_C	414
	SlDEAD41	DEAD-helicase_C	395
	SlDEAD42	DEAD-helicase_C	1148
ORYZA SATIVA	LOC_Os01g07080	DEAD-helicase_C-DUF4217	648
	LOC_Os01g07740	DEAD-helicase_C	760
	LOC_Os01g08930	DEAD-helicase_C	626
	LOC_Os01g10050	DEAD-helicase_C	495
	LOC_Os01g36860	DEAD-helicase_C	793
	LOC_Os01g43120	DEAD-helicase_C	595
	LOC_Os01g43130	DEAD-helicase_C	537
	LOC_Os01g45190	DEAD-helicase_C	405
	LOC_Os01g68320	DEAD-helicase_C	667
	LOC_Os02g05330	DEAD-helicase_C	415
	LOC_Os02g05660	DEAD-helicase_C	628
	LOC_Os02g12840	DUF4283-DEAD-helicase_C	882
	LOC_Os02g42860	DEAD-helicase_C	509
	LOC_Os02g57980	DEAD-helicase_C	812
	LOC_Os03g06220	DEAD-helicase_C	506
	LOC_Os03g19530	DEAD-helicase_C	771
	LOC_Os03g36930	DEAD-helicase_C	405
	LOC_Os03g46610	DEAD-helicase_C	473
	LOC_Os03g50090	DEAD-helicase_C	737
	LOC_Os03g51900	DEAD-helicase_C	671
	LOC_Os03g58810	DEAD-helicase_C-DUF4217	591
	LOC_Os03g59050	DEAD-helicase_C	638
	**LOC_Os03g61220**	DEAD-helicase_C-GUCT-Zf_CCHC	759
	LOC_Os04g43140	DEAD-helicase_C	833
	LOC_Os04g45040	DEAD-helicase_C	499
	LOC_Os05g01990	DEAD-helicase_C-DUF4217	592
	LOC_Os06g33520	DEAD-helicase_C	924
	LOC_Os06g40020	DEAD-helicase_C	479
	LOC_Os06g48210	DEAD-helicase_C	620
	LOC_Os06g48750	DEAD-helicase_C	415
	LOC_Os07g05050	DEAD-helicase_C	603
	LOC_Os07g10250	DEAD-helicase_C	639
	LOC_Os07g20580	DEAD-helicase_C	513

	LOC_Os07g33340	DEAD-helicase_C-DUF4217	773
	LOC_Os07g43980	DEAD-helicase_C	502
	LOC_Os07g46580	DEAD-DEAD-helicase_C	445
	LOC_Os08g05810	DEAD-helicase_C	948
	LOC_Os08g06344	DEAD-helicase_C	1050
	LOC_Os08g32090	DEAD-helicase_C-DBP10CT	852
	LOC_Os09g21520	DEAD-helicase_C	404
	**LOC_Os09g34910**	DEAD-helicase_C-GUCT	697
	LOC_Os10g35990	DEAD-helicase_C	471
	LOC_Os11g38670	DEAD-helicase_C	624
	LOC_Os11g46240	DEAD-helicase_C	1399
	LOC_Os12g29660	DEAD-helicase_C	803
	LOC_Os12g41715	DEAD-helicase_C	629
PHYSCOMITRELLA PATENS	**Pp3c10_20840**	DEAD-helicase_C-GUCT	696
	Pp3c11_17170	DEAD-helicase_C	471
	Pp3c11_22980	DEAD-helicase_C	414
	Pp3c1_12370	DEAD-helicase_C	583
	Pp3c11_24910	WW-DEAD-helicase_C	733
	Pp3c11_500	DEAD-helicase_C-DUF4217	631
	Pp3c1_21470	DEAD-helicase_C	938
	Pp3c1_22540	DEAD-helicase_C-DUF4217	619
	Pp3c1_24600	DEAD-helicase_C	615
	Pp3c1_24700	DEAD-helicase_C	540
	Pp3c1_37280	DEAD-helicase_C	453
	Pp3c13_7380	DEAD-helicase_C	603
	**Pp3c1_40060**	DEAD-helicase_C-GUCT-Zf_CCHC	746
	Pp3c1_40410	DEAD-helicase_C	635
	Pp3c14_17810	DEAD-helicase_C	636
	Pp3c14_6810	DEAD-helicase_C-DBP10CT	823
	Pp3c16_16520	DEAD-helicase_C	483
	Pp3c17_13450	DEAD-helicase_C-DUF4217	626
	Pp3c17_17420	DEAD-helicase_C	617
	Pp3c17_22010	DEAD-helicase_C	1050
	**Pp3c20_20710**	DEAD-helicase_C-GUCT	693
	Pp3c21_10710	DEAD-helicase_C	754
	Pp3c2_13290	DEAD-helicase_C	589
	Pp3c21_4750	DEAD-helicase_C	550
	Pp3c2_16950	DEAD-helicase_C	933
	Pp3c2_17270	DEAD-helicase_C-DUF4217	640
	Pp3c2_1920	DEAD-helicase_C	637
	Pp3c2_24110	DEAD-helicase_C	489
	Pp3c2_25070	DEAD-helicase_C	583

	Pp3c2_25130	DEAD-helicase_C	583
	**Pp3c2_2520**	DEAD-helicase_C-GUCT-Zf_CCHC	831
	Pp3c22_7840	DEAD-helicase_C	599
	**Pp3c2_2840**	DEAD-helicase_C-GUCT-Zf_CCHC	744
	Pp3c2_34920	DEAD-helicase_C	917
	Pp3c25_520	DEAD-helicase_C	617
	Pp3c27_1430	DEAD-helicase_C	593
	**Pp3c27_1440**	DEAD-helicase_C-GUCT-Zf_CCHC	742
	Pp3c4_14810	DEAD-helicase_C-DUF4217	865
	Pp3c5_17030	DEAD-helicase_C	1048
	Pp3c5_28210	DEAD-helicase_C	414
	Pp3c6_1080	DEAD-helicase_C	414
	Pp3c6_15830	DEAD-helicase_C	897
	Pp3c7_14910	DEAD-helicase_C	494
	Pp3c7_3770	DEAD-helicase_C	414
	Pp3c7_8530	WW-DEAD-helicase_C	727
	Pp3c8_1190	WW-DEAD-helicase_C	498
	Pp3c9_14310	DEAD-helicase_C	823
MARCHANTIA POLYMORPHA	Mapoly0001s0280	DEAD-helicase_C	502
	Mapoly0001s0493	DEAD-helicase_C	936
	Mapoly0002s0338	DEAD-helicase_C	670
	Mapoly0003s0204	DEAD-helicase_C	572
	Mapoly0003s0297	DEAD-helicase_C-DUF4217	631
	Mapoly0006s0271	DEAD-helicase_C	555
	Mapoly0008s0193	DEAD-helicase_C-DUF4217	857
	Mapoly0009s0142	DEAD-helicase_C	516
	Mapoly0011s0206	DEAD-helicase_C	975
	Mapoly0015s0089	DEAD-helicase_C	660
	Mapoly0020s0052	WW-DEAD-helicase_C	780
	**Mapoly0021s0161**	DEAD-helicase_C-GUCT	689
	Mapoly0027s0149	DEAD-helicase_C-DUF4217	622
	**Mapoly0039s0077**	DEAD-helicase_C-GUCT-Zf_CCHC	834
	Mapoly0043s0067	DEAD-helicase_C	1243
	Mapoly0046s0081	DEAD-helicase_C	628
	Mapoly0059s0052	DEAD-helicase_C	598
	Mapoly0062s0065	DEAD-helicase_C	620
	Mapoly0075s0037	DEAD-helicase_C	414
	Mapoly0076s0020	DEAD-helicase_C	872
	Mapoly0090s0088	DEAD-helicase_C	805
	Mapoly0095s0002	DEAD-helicase_C	580
	Mapoly0116s0020	DEAD-helicase_C	454
	Mapoly0122s0011	DEAD-helicase_C	585

	Mapoly0133s0003	DEAD-helicase_C	909
	Mapoly0164s0014	DEAD-helicase_C	592
	Mapoly0165s0019	DEAD-helicase_C-DBP10CT	795
	Mapoly0167s0009	DEAD-helicase_C-DUF4217	662
MESOSTIGMA VIRIDE	**Mv15069-RA**	DEAD-helicase_C-GUCT	657
	**Mv05255-RA**	DEAD-helicase_C-GUCT	845
	Mv14253-RA	DEAD-helicase_C	524
	Mv11439-RA	DEAD-helicase_C	565
	Mv05974-RA	DEAD-helicase_C	496
	Mv10354-RA	DEAD-helicase_C	650
	Mv14910-RA	DEAD-helicase_C	478
	Mv19187-RA	DEAD-helicase_C	689
	Mv17563-RA	DEAD-helicase_C	1036
	Mv09192-RA	DEAD-helicase_C	440
	Mv09769-RA	DEAD-helicase_C	719
	Mv11662-RA	DEAD-helicase_C	487
	Mv06156-RA	DEAD-helicase_C	769
	Mv14240-RA	DEAD-helicase_C-DUF4217	588
	Mv04709-RA	DEAD-helicase_C	401
	Mv22670-RA	DEAD-helicase_C-DUF4217	623
	Mv15167-RA	DEAD-helicase_C	985
	Mv19190-RA	DEAD-helicase_C-DUF4217	980
	Mv12277-RA	DEAD-helicase_C	737
	Mv06837-RA	DEAD-helicase_C	363
	Mv05536-RA ★	DEAD	469
	Mv01782-RA	DEAD-helicase_C	562
	Mv12289-RA	DEAD-helicase_C	706
	Mv06634-RA	DEAD-helicase_C	1039
	Mv22148-RA ★	DEAD/SPRY-helicase_C	742
	Mv18390-RA	DEAD-helicase_C	611
	Mv21769-RA	DEAD-helicase_C	586
	Mv06028-RA	DEAD-helicase_C	689
	Mv03699-RA	DEAD-helicase_C	302
	Mv17227-RA	DEAD-helicase_C	526
	Mv08303-RA	DEAD-helicase_C	863
	Mv17533-RA ★	DEAD-helicase_C	880
	Mv15810-RA ★	DEAD-helicase_C-DUF4217	1171
	Mv12435-RA ★	DEAD-helicase_C-Dicer_Dimer	3291
	Mv11178-RA	DEAD-helicase_C	614

CHLAMYDOMONAS REINHARDTII	Cre01.g021600	WW-DEAD-helicase_C	624
	Cre01.g022350 ★	DEAD-helicase_C	1460
	Cre01.g028200	DEAD-helicase_C-helicase_C	751
	Cre01.g033832	DEAD-helicase_C	616
	Cre02.g090500	DEAD-helicase_C	1257
	Cre02.g141000 ★	DEAD-helicase_C-Dicer_Dimer	2237
	Cre03.g156150	DEAD-helicase_C-DUF4217	911
	Cre03.g158750	DEAD-helicase_C	571
	Cre03.g166650	DEAD-helicase_C	666
	Cre03.g188550	DEAD-helicase_C-DUF4217	914
	Cre03.g188750 ★	DEAD/SPRY-helicase_C	776
	Cre03.g199647	DEAD-helicase_C	400
	Cre03.g200095	DEAD-helicase_C	935
	Cre04.g223850	DEAD-helicase_C	406
	Cre05.g238900	DEAD-helicase_C	771
	Cre06.g282600	DEAD-helicase_C-DUF4217	737
	Cre06.g291150	DEAD-helicase_C	819
	Cre06.g298650	DEAD-helicase_C	465
	Cre06.g306850	DEAD-helicase_C	505
	Cre07.g314900	DEAD-helicase_C-DUF4217	732
	Cre07.g334200	DEAD-helicase_C	645
	Cre07.g349300	DEAD-helicase_C	879
	Cre10.g420900	DEAD-helicase_C	930
	Cre10.g427700	DEAD-helicase_C	647
	Cre12.g505200	DEAD-helicase_C	447
	Cre12.g522850	DEAD-helicase_C	1037
	Cre12.g526850	DEAD-helicase_C	714
	Cre12.g539100	DEAD-helicase_C	624
	Cre12.g540200	DEAD-helicase_C	1118
	Cre13.g592150	DEAD-helicase_C	908
	Cre16.g661900	DEAD-helicase_C	1448
	Cre16.g662000	DEAD-helicase_C	653
	Cre16.g676400	DEAD-helicase_C	1426
	Cre16.g683500	DEAD-helicase_C	547
	**Cre17.g727700**	DEAD-helicase_C-GUCT	827
	Cre17.g744147	PWI-DEAD-helicase_C-Sec63-DEAD	2169

Sequences marked with a star (★) were omitted from the phylogenetic analysis. GUCT containing sequences are shown in bold. aa: amino acids.

Supplementary Table S3

Oligonucleotide primers and their sequences used in the present study.

Purpose	Primer name	Sequence (5’ to 3’)
Cloning	SP_5’_ARH1	TTCGAACGTACGTCGAATCCACCCATCTTAACCATCATG
	ASP_5’_ARH1	AGTTATCTCGAGTCGCTTTGATGACGAAGAAACCTCC
	SP_3’_ARH1	ACGAAGTTATACTAGCTCAAAAGTGAAGACACCAAATATG
	ASP_3’_ARH1	GCGTGGCGCCACTAGAGGTGCTTTGTCTTATGGAAATTC
	SP_5’_ARH2	TTCGAACGTACGTCGTTGTGAAAGCATGGAGTGTAATG
	ASP_5’_ARH2	AGTTATCTCGAGTCGTCTCACCGAAGTAAATTCTCAAGAC
	SP_3’_ARH2	CGCCACGCGTGATATTGACGTCTACTTCTGTTGGCC
	ASP_3’_ARH2	ATGTTAACATGCATGGGACCCACCCAATCACAAAATG
	pBHRF-5In-SP	CGACTCGAGATAACTTCGTATAATG
	pBHRF-5In-ASP	CGACGTACGTTCGAACAATT
	pBHRF-1-SP	CATGCATGTTAACATCGATCCATGG
	pBHRF-1-ASP	ATATCACGCGTGGCGCCACTAG
	SP_5’-KI	GATTGTGTGAGCTGTATGATAGTG
	ASP_5’-KI	AGAGCCTCCGCCTCCACGACGACCAAACCGTC
	SP_5’Inf-KI	TTCGAACGTACGTCGGATTGTGTGAGCTGTATGATAGTGG
	ASP_5’Inf-KI	ACTTTTGCTAGCTCGccAGAGCCTCCGCCTCCACG
	SP_3’Inf-KI	ACGAAGTTATACTAGTTTCTTTCTTTTGACCTCCATTT
	ASP_3’Inf-KI	GCGTGGCGCCACTAGGAAGGTGCTTTGTCTTATGGAAA
	nru-nhe-meGFP-fw	TCGCGAGCTAGCACAAAAGTTGTGGGATCCATG
	xho-meGFP-rev	CTCGAGACTTTGTATAGAAAAGTTGGGTGTTAC
Genotyping	F-KO1_GS	GAAAAAAGAGAAGAAGGTGG
	R-KO1_GS	TGGAGTCATGTGCGGTAA
	F-KO1_GT	ATATAGGAAAATACAAAATAGATCCAAGT
	R-KO1_GT	TGATGGTCATACAATTTTGTTGTTG
	F-KO2_GS	CGAAGAAGAAGAGAGCCAA
	R-KO2_GS	ACCACAATATCAACACCCC
	F-KO2_GT	CAGTAGAGAGTGTCGAAA
	R-KO2_GT	TTGGGACCTGAGGGGATCAT
	F-KI_GS	GACAGGAAGAAGGAGTACA
	R-KI_GS	CACCTCTTACAACCATCAC
	F-KI_GT	ATATGGGGTTTGTGGACGA
	R-KI_GT	ATTCTCACTTCCAACACC
	35S-R	TAAAGTGACAGATAGCTGGG
	Term-F	AGGGTTCTTATAGGGTTTCGCTCATG
q-PCR	Arh1q-F	CTCGGAGTTGGCAGGAGTCT
	Arh1q-R	AGGAGGTTCACACTGAATCACCAAC
	Arh2q-F	CTCAGAACTCGCAGGAGCTC
	Arh2q-R	GAGGCTCACACTGAATCACCAGT
	RSBq-F	GTCGACGAGCAGTCGAAGAA
	RSBq-R	ACTTCTGGAACCGAGAACGG
Southern hybridization probes	5’TGSp-F	TTCGAACGTACGTCGTTGTGAAAGCATGGAGTGTAATG
	5’TGSp-R	AGTTATCTCGAGTCGTCTCACCGAAGTAAATTCTCAAGAC
	3’TGSp-F	CGCCACGCGTGATATTGACGTCTACTTCTGTTGGCC
	3’TGSp-R	ATGTTAACATGCATGGGACCCACCCAATCACAAAATG

Supplementary Results S1

Establishment of the P. patens knock-in mutant rh1-3xgfp

In order to evaluate the cellular localization of *Pp*RH1, we generated the *P. patens rh1-3xgfp* strain containing a green fluorescent protein (GFP) triplicate tag in C-terminal position, using homologous recombination (Supplementary Fig. S3, a). This was achieved by polyethylene glycerol-mediated transformation of *P. patens* protoplasts with knock-in vector phel_GFP_KI (Supplementary Fig. S14, a). 13 stable transformants were isolated. Of these, three individual strains displayed a single copy insertion pattern by PCR. The three PCR positive lines were indistinguishable both morphologically and in term of life cycle from wild-type plants. Finally, specific investigation by confocal microscopy showed a similar fluorescence (GFP) pattern in these lines. One of these lines is presented in this study as *rh1-3xgfp*. Polymerase chain reaction (PCR) genotyping of *rh1-3xgfp* showing targeted single-copy insertion 3' to *PpRH1* is provided in Supplementary Fig. S3, b.

Establishment of the P. patens deletion mutants Δrh1, Δrh2 and Δrh1/2

To study the function of the *Pp*RH1 and *Pp*RH2 proteins, we generated the single deletion mutants *Δrh1* and *Δrh2,* respectively by homologous recombination. Polyethylene glycerol-mediated transformation of *P. patens* protoplasts with knockout construct pBHRF_hel1_KO and pBNRF_hel2_KO (Supplementary Fig. S14, b,c) was conducted to generate the *Δrh1* and *Δrh2* mutants, respectively. After transformation 82 putative *Δrh1* and 90 putative *Δrh2* stable transformants were identified. These transformants were subjected to three rounds of PCR genotyping identifying 10 *Δrh1* and 7 *Δrh2* individuals harboring loss of locus and the appropriate 5' and 3' targeting events. The *Δrh1* individuals did not display any phenotype whatsoever, and one was chosen as the working strain and termed *Δrh1.* The individual *Δrh2* plants presented minimal protonemata defects, and one was chosen as the working strain and termed *Δrh2.*

To analyze the functional redundancy of the *PpRH*1 and *PpRH2* genes, we also generated the *Δrh1/2* double knockout line by transforming *Δrh2/cre* protoplasts*,* a marker-free *Δrh2* line generated by cre/lox-mediated excision of the antibiotic resistant

cassette (Supplementary Fig. S5, c) with pBHRF_hel1_KO (Supplementary Fig. S14, b), yielding 27 stable transformants of which 9 were PCR genotyping positive (loss of locus and appropriate 5’ and 3’ targeting). These 9 lines displayed a clear protonemal growth reduction. One of the strains was chosen as the working strain and termed *Δrh1/2.* Supplementary Fig. S5, d presents the PCR genotyping results of *Δrh1*, *Δrh2* and *Δrh1/2*, confirming deletion of *PpRH*1 and *PpRH2* in the corresponding single and double mutant lines in addition to the appropriate 5’ and 3’ targeting events. Southern Blot hybridization confirmed the *Δrh2/cre* mutant (Supplementary Fig. S5, e). Quantitative reverse transcription PCR (RT-qPCR) (Fig. 2b) and RT-PCR (Supplementary Fig. S5, f) revealed no detectable levels of *PpRH1* and *PpRH2* transcripts in the corresponding knockout mutants confirming the targeted removal of the genes.

Gametophyte growth under stress conditions

Low light conditions (5 μmol.m^{-2}.s^{-1}) caused significant reduction of protonemata spreading with calculated plant area around 15 mm^2 in WT and *Δrh1* (Supplementary Fig. S7, a) compared to 65 mm^2 under standard conditions. Slowly growing protonemata were predominantly composed of chloronema cells. Side branch growth was increased in *Δrh2* to the point that they overgrow slowly expanding primary filaments (Supplementary Fig. S7, b,c).

High light conditions (140 μmol.m^{-2}.s^{-1}) affected primary filaments extension, regularity of side-branching and stimulated gametophore growth. Nevertheless, enhanced elongation of side branches in *Δrh2* and their strong reduction in *Δrh1/2* compared to WT and *Δrh1* remained consistent (Supplementary Fig. S8).

Salt stress reduced gametophore formation but on the other hand stimulated protonemata growth. Inoculated plants appeared bushier compared to standard conditions, with actively branching secondary filaments. Again, side branch extension was visibly increased in *Δrh2* and strongly reduced in *Δrh1/2* when compared to WT and *Δrh1* (Supplementary Fig. S9 b,c).

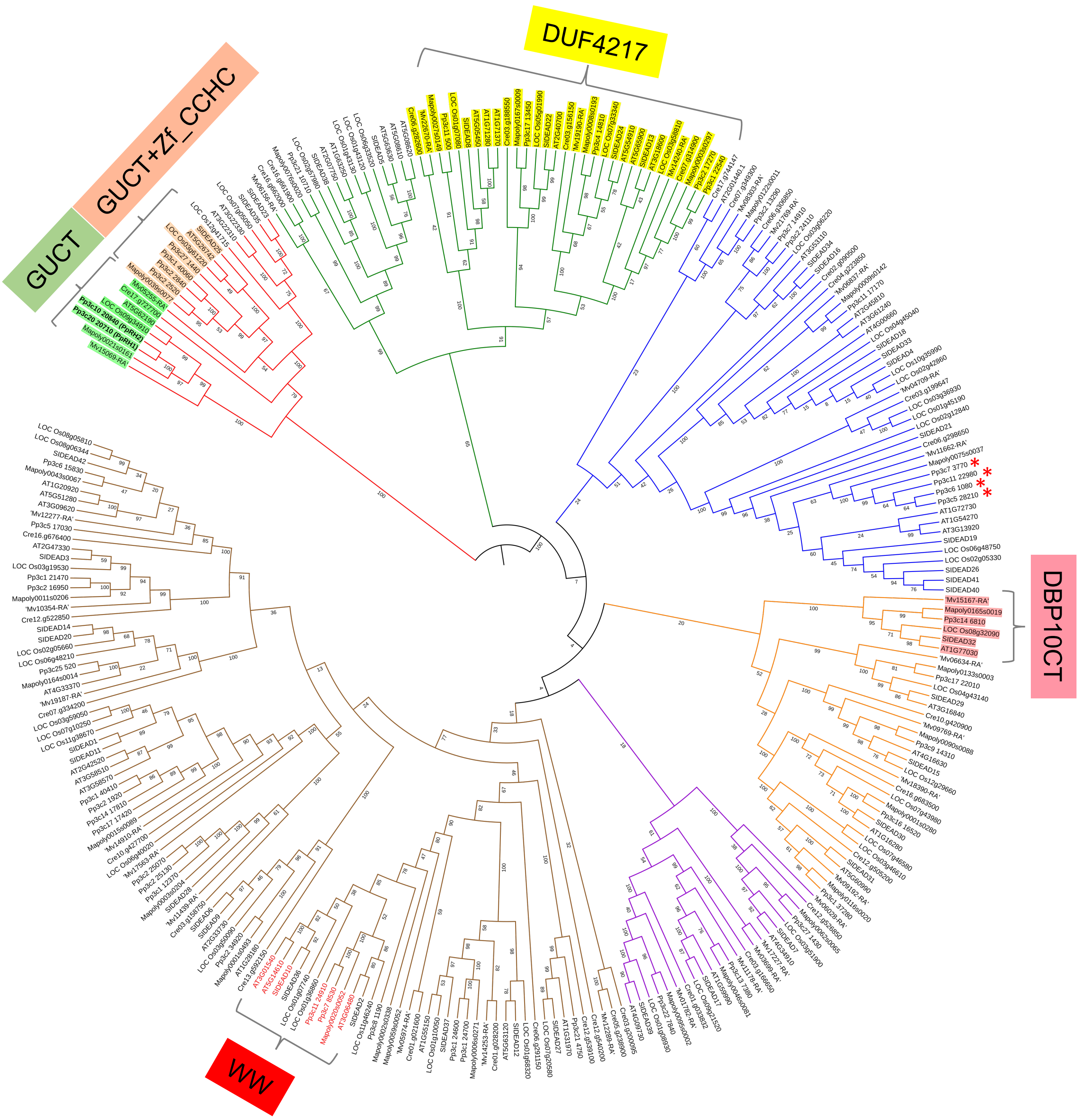

Fig S1 Phylogenetic analysis of the core domain of DEAD-box RNA helicases in *Arabidopsis thaliana*, *Oryza sativa*, *Solanum lycopersiucum*, *Physcomitrium patens*, *Marchantia polymorpha*, *Mesostigma viride* and *Chlamydomonas reinhardtii*. The RNA helicases cluster into six major clades (shown in different colors). Proteins containing the GUCT, GUCT-Zf-CCHC, WW, DUF4217 or DBP10CT domains each clustered separately into one individual group. The eukaryotic translation initiation factors (eIF4A) proteins in *P. patens* are shown with a red star (Tyagi et al., 2020). The sequences listed without a black star (★) in Supplementary Table S2 are included in this analysis. The sequences were aligned with MAFFT (v.7) and E-Ins-I. Phylogenetic reconstructions was performed using the neighbor-joining method in the CLC genomic workbench v. 12 with 1000 bootstrap iterations.

(A)

PpRH1

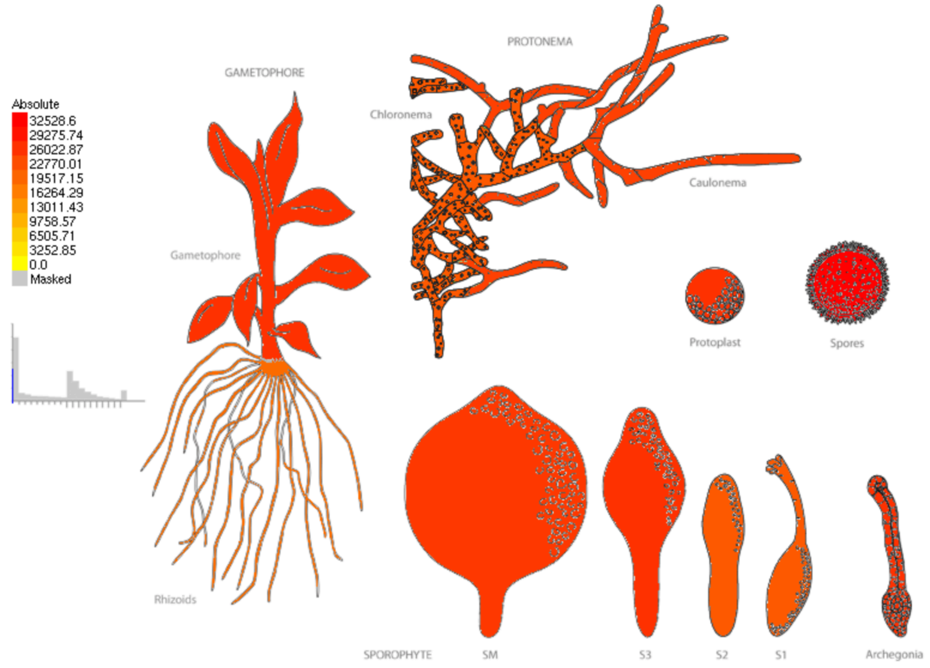

(B)

PpRH2

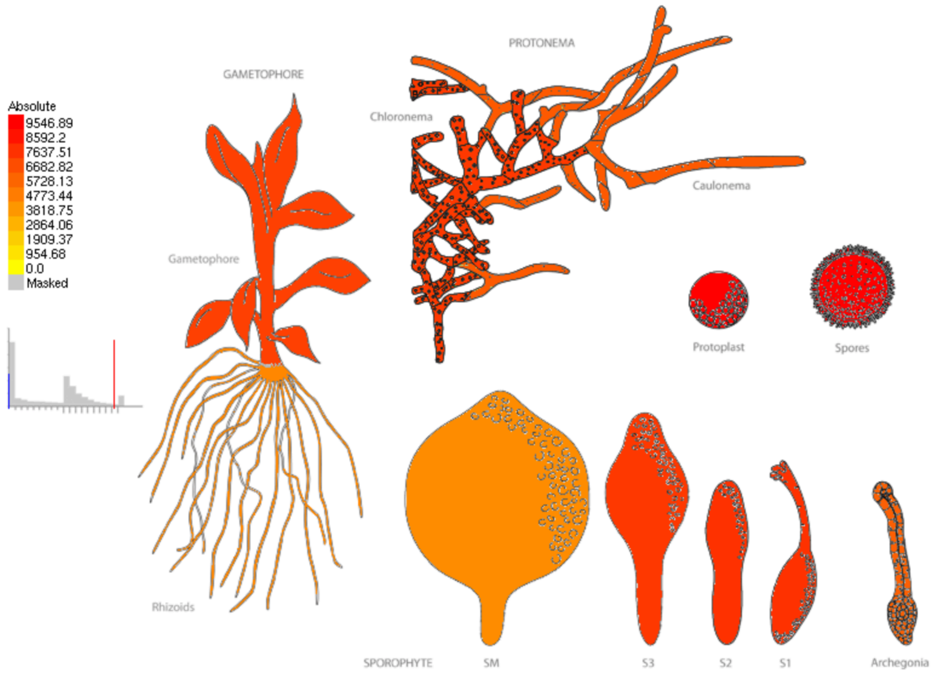

Fig S2 *PpRH1* and *PpRH2* expression profiles in *Physcomitrium patens* tissue. The absolute expression level of **a** *PpRH1* (Pp3c20_20710) and **b** *PpRH2* (Pp3c10_20840) using color codes as reported by the Physcomitrella eFP browser, showing the ubiquitous expression of both genes and the higher expression of *PpRH2* relative to *PpRH1.*

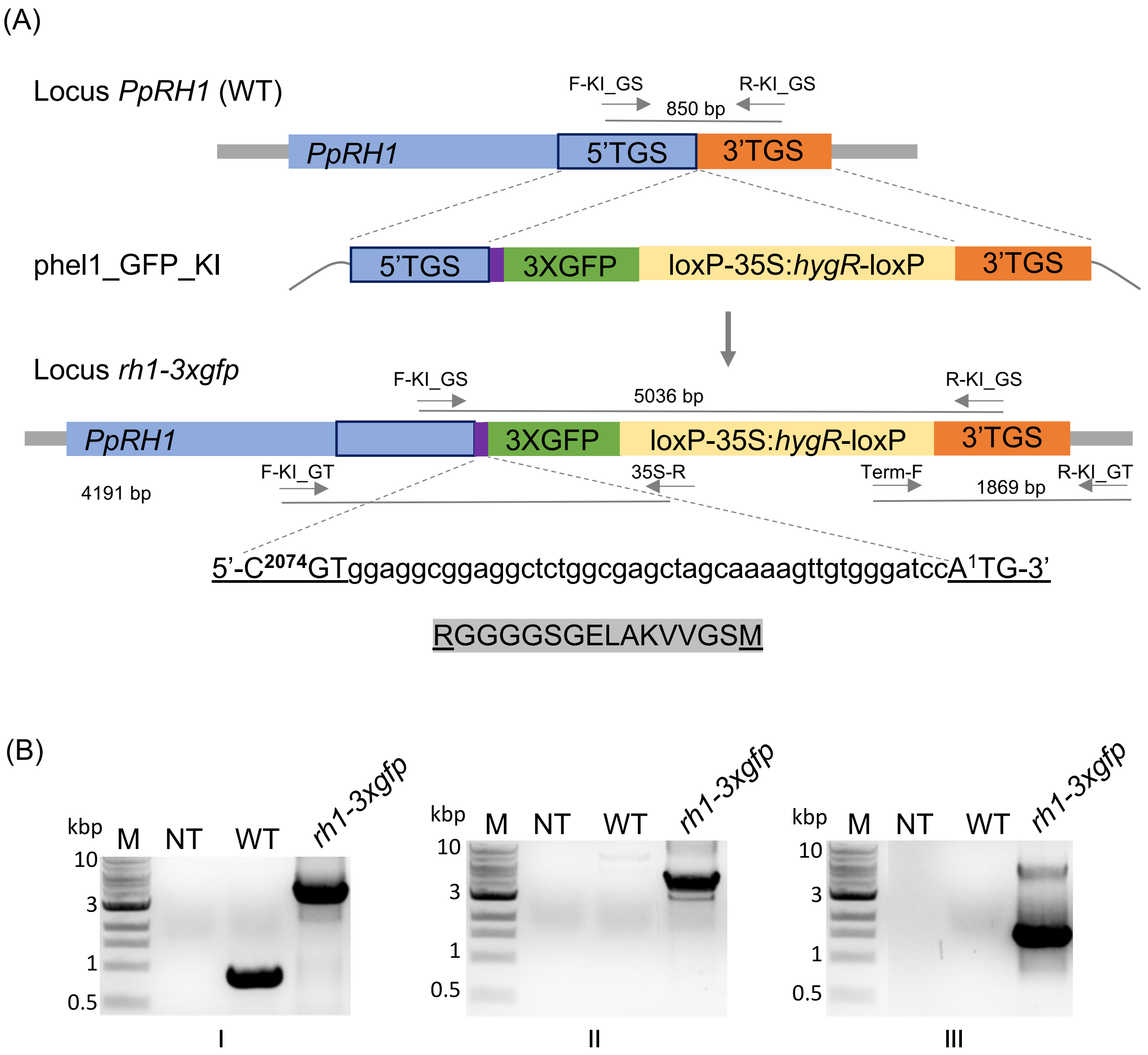

Fig S3 Targeted insertion and PCR genotyping of the *P. patens rh1-3xgfp* mutant line. **a** Schematic representation of the GFP knock-in strategy to create the *rh1-3xgfp* mutant using vector phe1_GFP_KI. The purple box between the 5'TGS and 3XGFP sequences represent the linker sequence between *PpRH1* and *GFP,* corresponding to the amino acid sequence provided in the grey box. The last (R) and first (M) amino acids in *Pp*RH1 and GFP, respectively are underlined. The corresponding nucleic acid sequence of the linker is shown above the amino acid sequence; the numbers 2074 and 1 refer to the nucleotide position in genes *PpRH1* and *GFP*, respectively. Annealing sites for primers used for PCR genotyping are shown with arrows (the sequences of the primers can be found in Supplemental Data S1). The expected PCR amplicons and their sizes are indicated with grey solid lines. **b** Gel-electrophoresis analysis showing the expected PCR product sizes for WT and *rh1-3xgfp* with primer set I (F-KI_GS/R-KI_GS), II (F-KI_GT/35S-R) and III (Term-F/R-KI_GT). M = DNA size marker; NT = non-template PCR control.

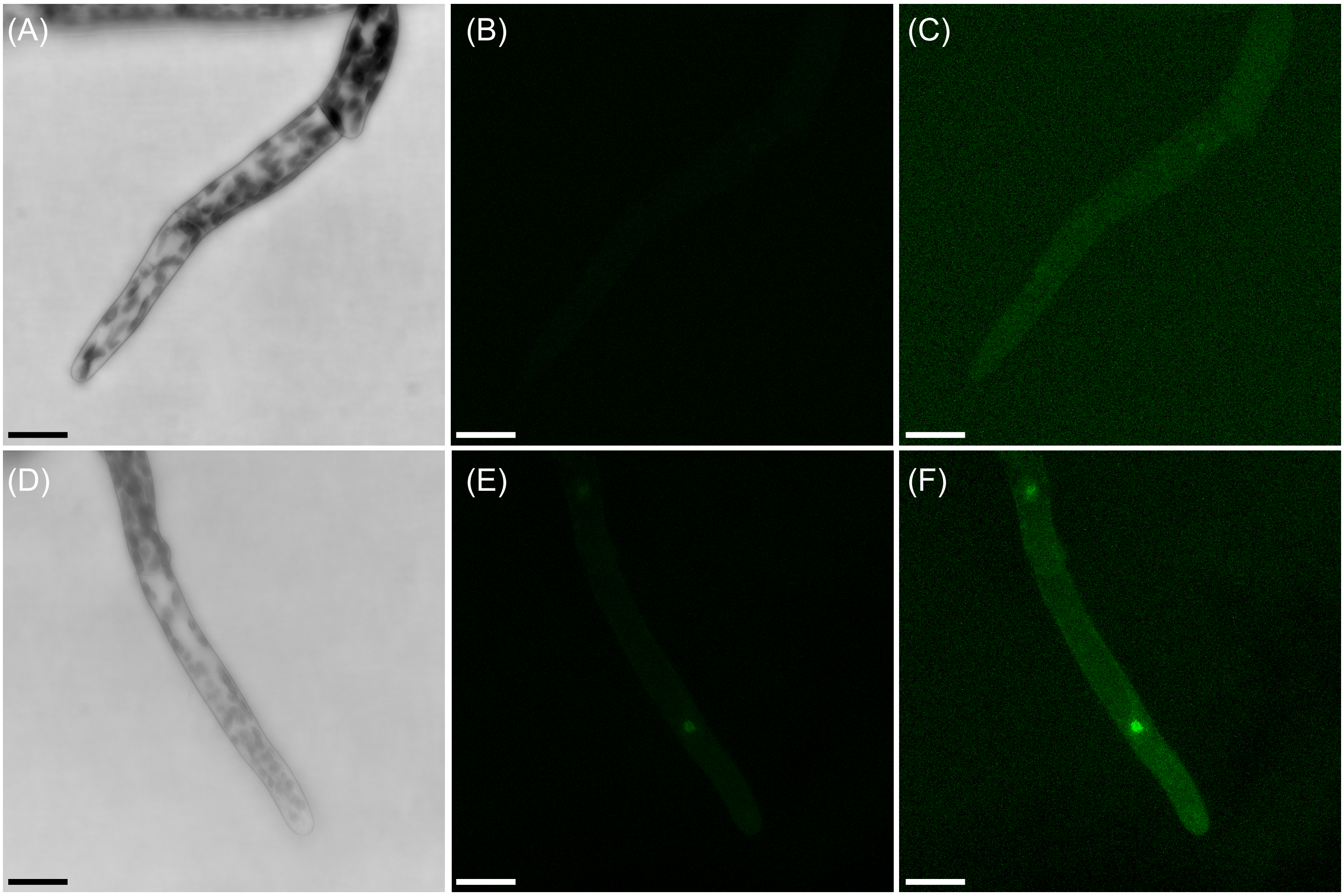

Fig S4 Comparison of the GFP fluorescent signal between *rh1-3xgfp* and wild-type protonema. a-c Tip cell of filament of wild-type *Physcomitrium patens* protonema. **d-f** Tip cell of filament of *rh1-3xgfp* protonema. **a** and **d** Average projection of confocal stack of transmitted light acquisition. **b** and **e** Maximum projection of confocal stack of PpRH1-3xGFP fluorescent signal without overexposure. **c** and **f** Maximum projection of confocal stack of PpRH1-3xGFP fluorescent signal with overexposure. PpRH1-3xGFP accumulates in nucleus, a signal absent from the wild-type strain. Bar: 25 µm.

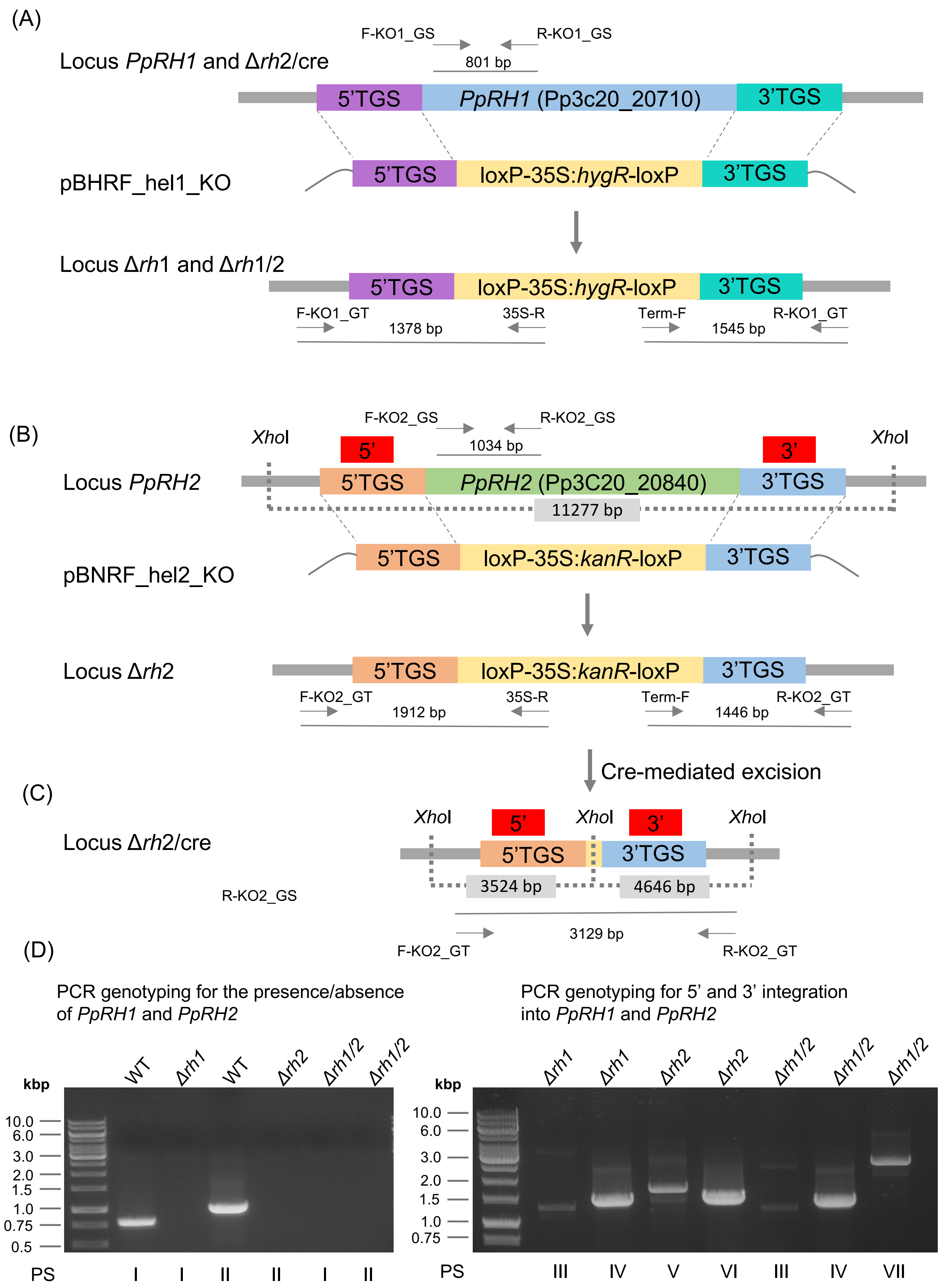

(A)
F-KO1_GS
R-KO1_GS
801 bp
Locus PpRH1 and Δrh2/cre
5'TGS
PpRH1 (Pp3c20_20710)
3'TGS
pBHRF_hel1_KO
5'TGS
loxP-35S:hygR-loxP
3'TGS
Locus Δrh1 and Δrh1/2
5'TGS
loxP-35S:hygR-loxP
3'TGS
F-KO1_GT
35S-R
Term-F
R-KO1_GT
1378 bp
1545 bp
(B)
F-KO2_GS
R-KO2_GS
1034 bp
XhoI
XhoI
5'
3'
Locus PpRH2
5'TGS
PpRH2 (Pp3C20_20840)
3'TGS
11277 bp
pBNRF_hel2_KO
5'TGS
loxP-35S:kanR-loxP
3'TGS
Locus Δrh2
5'TGS
loxP-35S:kanR-loxP
3'TGS
F-KO2_GT
35S-R
Term-F
R-KO2_GT
1912 bp
1446 bp
Cre-mediated excision
(C)
Locus Δrh2/cre
XhoI
XhoI
XhoI
5'
3'
5'TGS
3'TGS
3524 bp
4646 bp
R-KO2_GS
3129 bp
F-KO2_GT
R-KO2_GT
(D)
PCR genotyping for the presence/absence of PpRH1 and PpRH2
PCR genotyping for 5' and 3' integration into PpRH1 and PpRH2
kbp
10.0
6.0
3.0
2.0
1.5
1.0
0.75
0.5
WT
Δrh1
WT
Δrh2
Δrh1/2
Δrh1/2
PS
I
I
II
II
I
II
kbp
10.0
6.0
3.0
2.0
1.5
1.0
0.75
Δrh1
Δrh1
Δrh2
Δrh2
Δrh1/2
Δrh1/2
Δrh1/2
PS
III
IV
V
VI
III
IV
VII

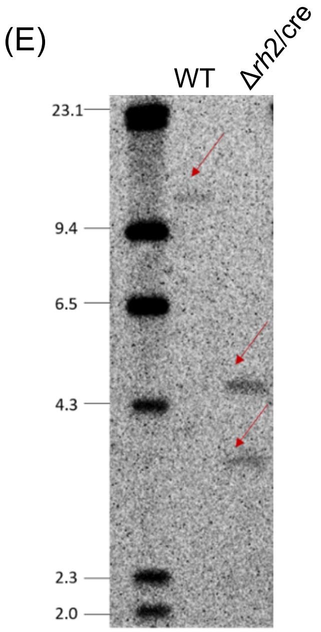

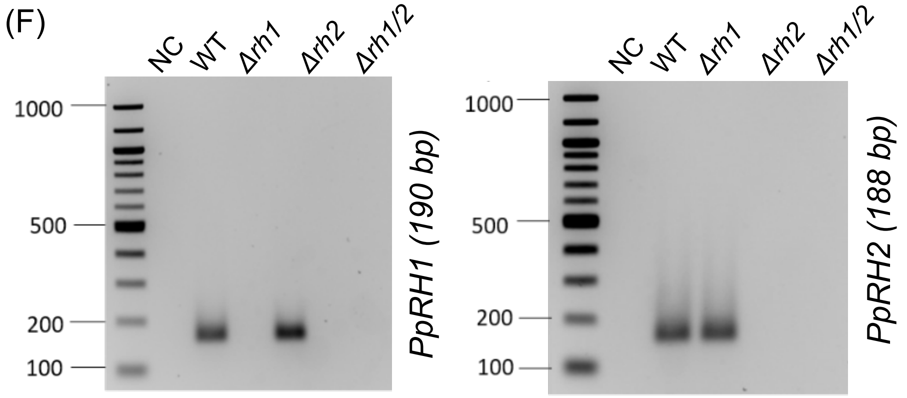

Fig S5 Targeted insertion and molecular characterization of the *P. patens* Δ*rh*1, Δ*rh*2, Δ*rh1/*2 and Δ*rh*2/cre mutant lines. a Schematic representation of the *PpRH1* gene-targeting strategy to make the Δ*rh*1 and Δ*rh*1/2 mutants by transformation of WT and *Δrh2/cre,* resepctively, using the pBHRF_hel1_KO plasmid vector. Annealing sites for primers used for PCR genotyping are shown with arrows and the sizes of the expected products are given above the solid grey line. **b** Schematic representation of the *PpRH2* gene-targeting strategy to make the *Δrh2* mutant by transformation of WT *P. patens* protoplasts with the pBHRF_hel2_KO plasmid vector, and **c** elimination of the resistant cassettes by Cre-mediated excision resulting in the establishment of the *Δrh2/cre* mutant. Annealing sites for primers used for PCR genotyping are shown with arrows and the sizes of the expected products are given above the solid grey line. The hybridization sites of the Southern blotting 5' and 3' probes are shown in red boxes. The restriction enzyme used for Southern blotting and the localization of the recognition sites are indicated. The sizes of the expected Southern Blot fragments are given in grey boxes. **d** PCR genotyping of the Δ*rh*1, Δ*rh*2 and Δ*rh1/*2 mutants. DNA was extracted from WT and mutant lines and used as templates in PCR reactions with the following primersetts (PS); I: F-KO1_GS/R-KO1_GS; **II**: F-KO2_GS/R-KO2_GS; **III**: F-KO1_GT/35S-R; IV: Term-F/R-KO1_GT; V: F-KO2_GT/35S-R; VI: F-KO2_GT/35S-R; VII: F-KO2_GT/R-KO2_GT. The sizes of the PCR products are in accordance with the expected sizes shown in a-c. **e** Southern blot analysis of the Δ*rh*2/cre mutant. The analysis was performed to confirm singel copy targeted insertion in the mutant and the elimination of the resistant cassette prior to transformation. Restriction fragments were generated using *XhoI*I and a blot hybridized with a mixture of 5' and 3' TGS probes displayed the expected hybridization signals. M = marker; WT = wild type. **f** RT-PCR analysis of *PpRH1* and PpRH2 expression in *P. patens* WT and Δ*rh*1, Δ*rh*2, Δ*rh1/*2 mutant plants. Amplicons of the expected sizes were detected in WT samples amplified with primers specific for both *PpRH1* and *PpRH2,* but were absent in the corresponding mutants. Primers used for amplification; for *PpRH1*: Arh1q-F/Arh1q-R; for *PpRH2*: Arh2q-F/ Arh2q-R. NC = non-template control.

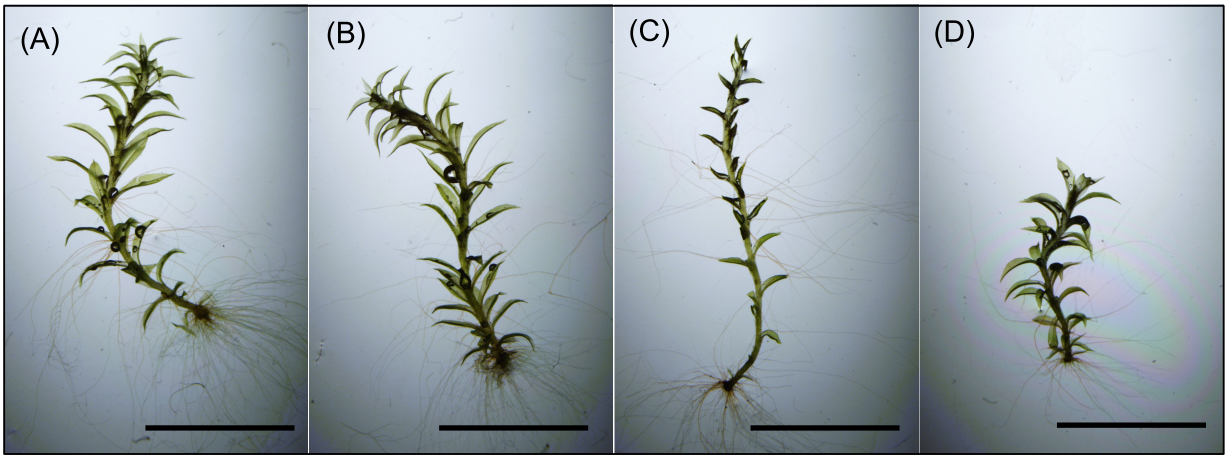

Fig S6 Long-term effect of reduced light on WT compared to *PpRH1* and *PpRH2* deletion mutant gametophores. Isolated gametophores from three month old cultures of WT, *PpRH1* deletion mutant (*Δrh1*), PpRH2 deletion mutant (*Δrh2*) and double deletion mutant (*Δrh1/2*). Cultures were cultivated on BCD medium under 16h light/8h dark cycle with light intensity 10 µmol.m-2.s-1 at 24°C. Scale bar: 1cm.

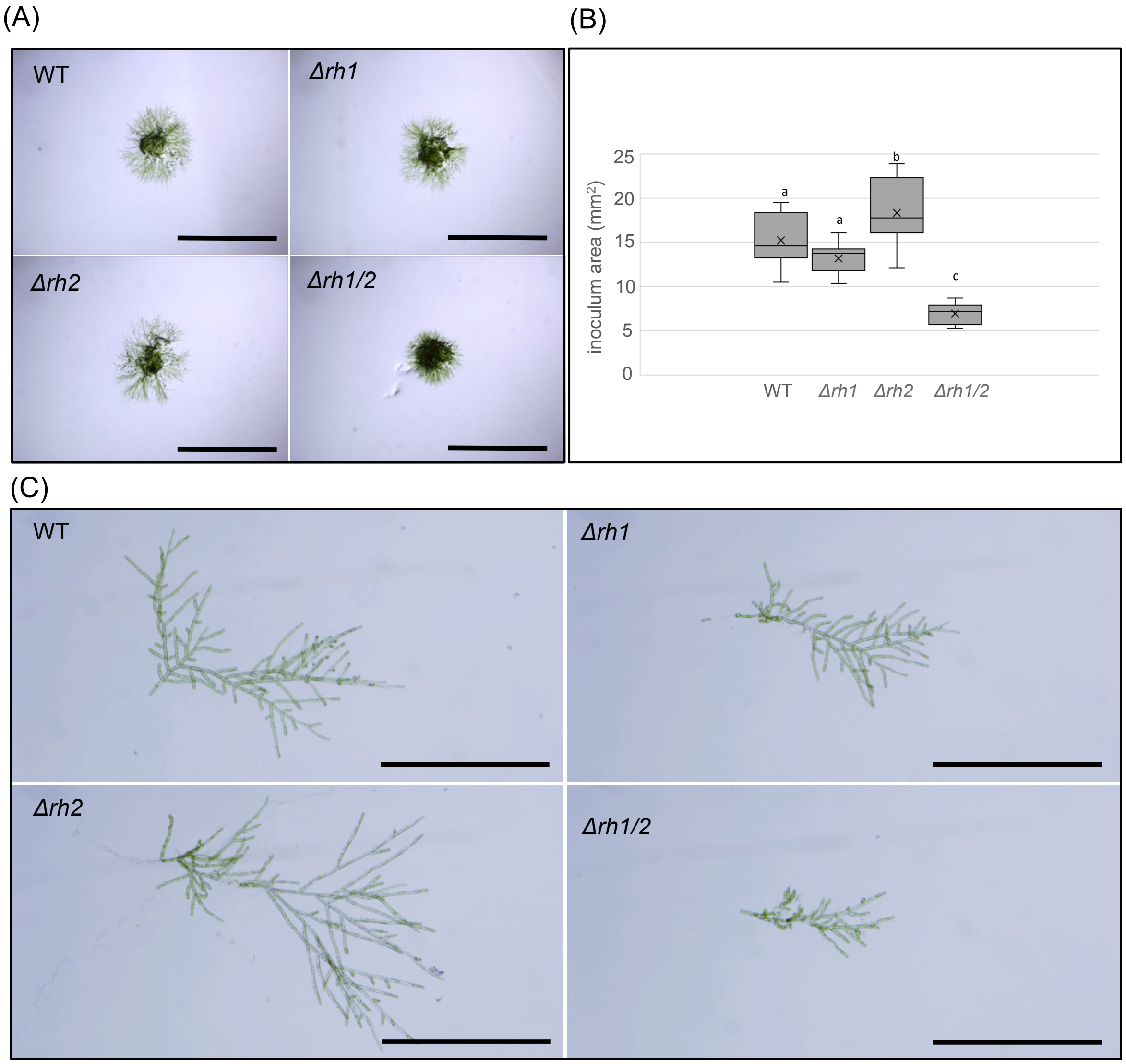

Fig S7 Vegetative growth under low light. a Two weeks old spot inocula grown on minimal BCD medium under low light (5 µmol.m-^{2}.s^{-1}, 16h light/8h dark at 24°C). **b** The graph represents the area of elipse surrounding individual inocula for each strain as measured and calculated using PROMICRA QuickPHOTO MICRO 3.0 software. Statistical significance (a, b, c) of the means was assessed at 95% confidence. Analysis of variance (ANOVA) and least significant difference (LSD) was performed in multiple sample comparison (n = 12). **c** Detailed view on isolated filaments shows accelerated side branch growth in *Δrh2* and reduced protonemata growth in *Δrh1/2* compared to WT and *Δrh1*. . Scale bars: 5,0 mm in **a**, 1,0 mm in **c**.

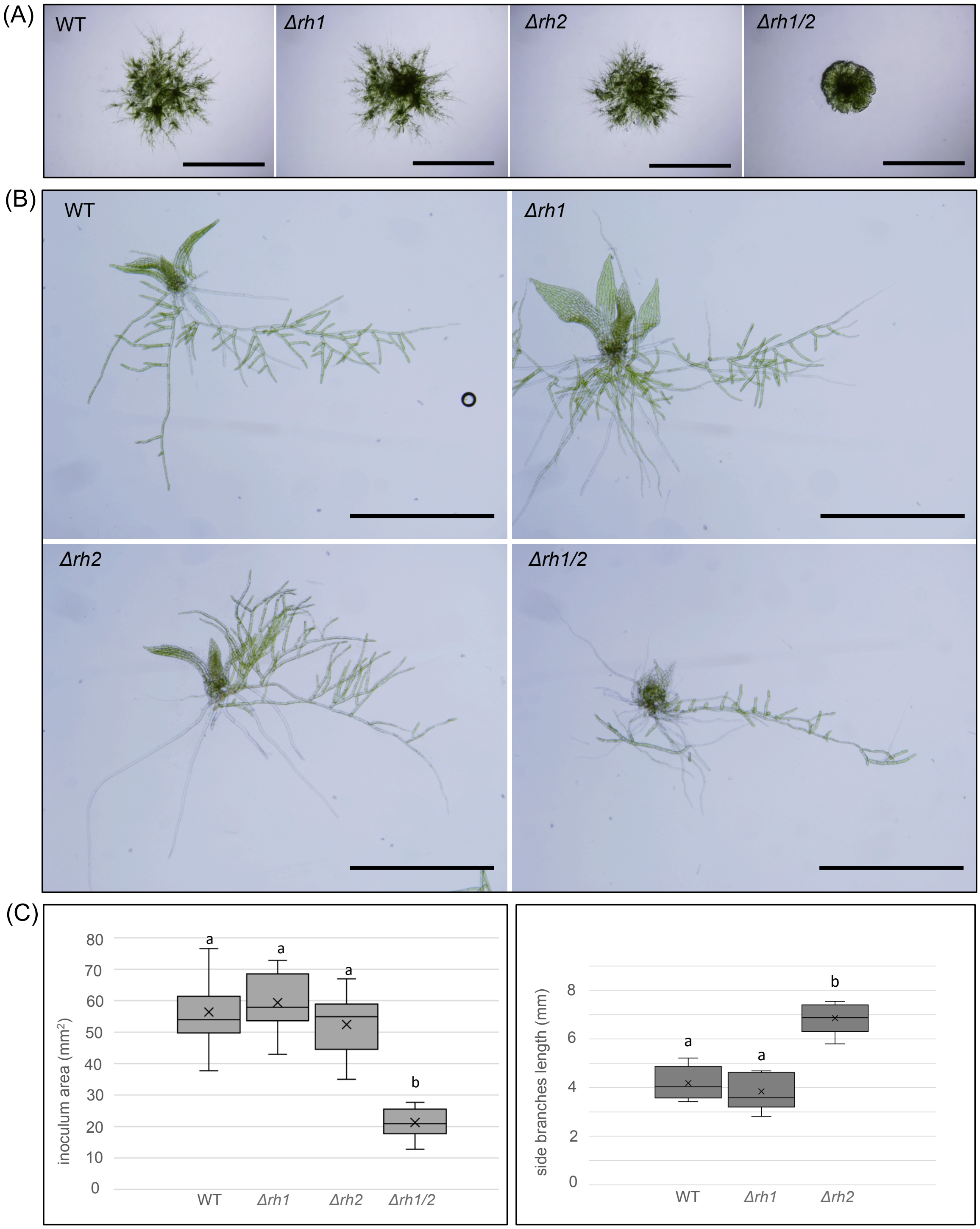

Fig S8 Vegetative growth under high light. a Two weeks old spot inocula grown on minimal BCD medium under high light (140 µmol.m^{-2}.s^{-1}, 16h light/8h dark at 24°C). **b** Detailed view on isolated filaments shows accelerated protonemata side branch growth in *Δrh2* and aberrant protonemata growth in *Δrh1/2* compared to WT and *Δrh1.* **c** Left graph represents the area of elipse surrounding individual inocula for each strain. Right graph: the length of first ten side branches (the sum) counted from the tip of the primary filament. Measurements were performed using PROMICRA QuickPHOTO MICRO 3.0 software. Statistical significance (a, b, c) of the means was assessed at 95% confidence. Analysis of variance (ANOVA) and least significant difference (LSD) was performed in multiple sample comparison (n = 12). Scale bars: 5,0 mm in **a**, 1,0 mm in **b**.

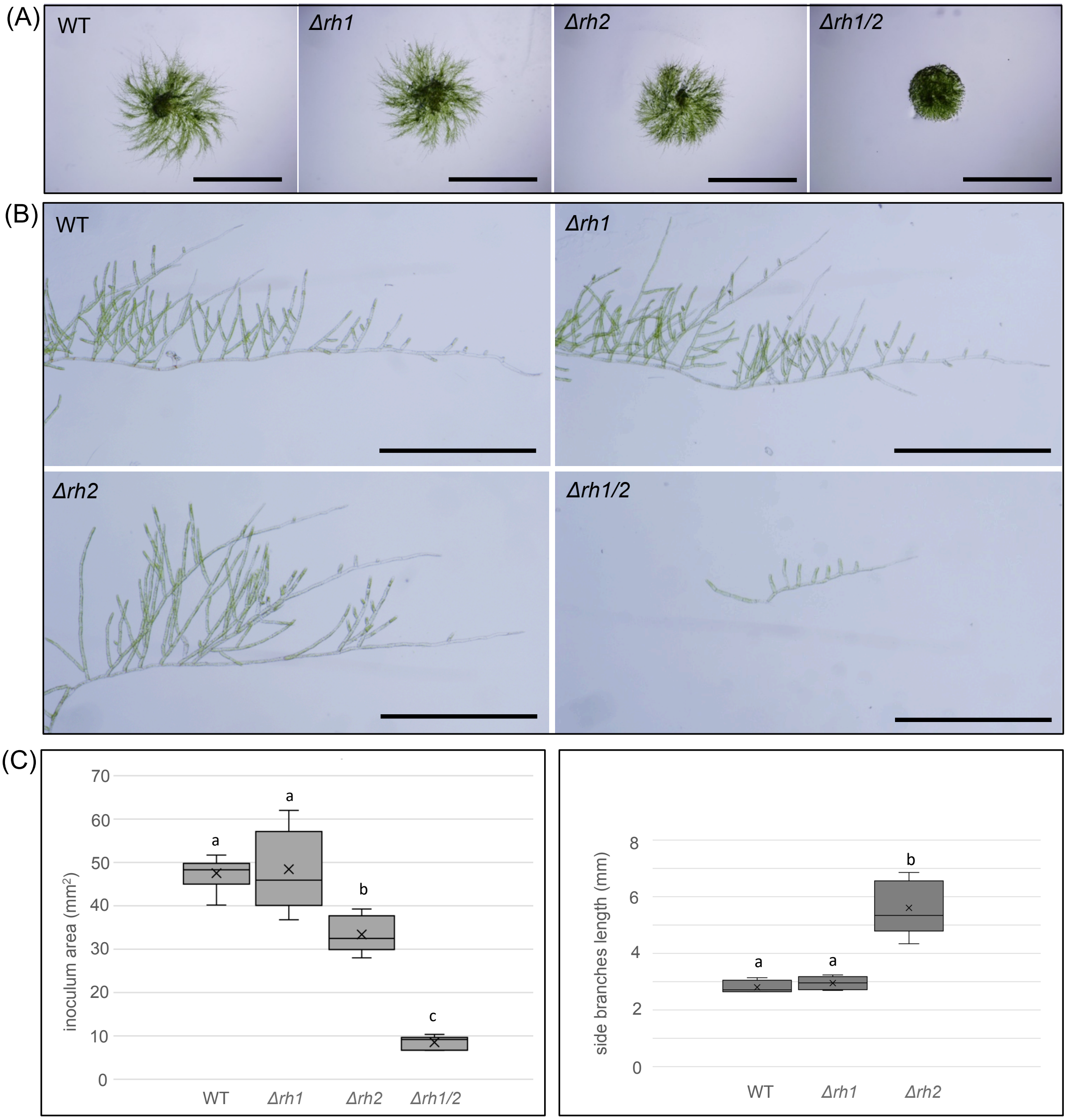

Fig S9 Vegetative growth on 90mM NaCl containing medium. a Two weeks old spot inocula grown on minimal BCD medium supplemented with 90mM NaCl. **b** Detailed view on isolated filaments shows accelerated protonemata side branch growth in *Δrh2* and aberrant protonemata growth in *Δrh1/2* compared to WT and *Δrh1.* **c** Left graph represents the area of elipse surrounding individual inocula for each strain. Right graph: the length of first ten side branches (the sum) counted from the tip of the primary filament. Measurements were performed using PROMICRA QuickPHOTO MICRO 3.0 software. Statistical significance (a, b, c) of the means was assessed at 95% confidence. Analysis of variance (ANOVA) and least significant difference (LSD) was performed in multiple sample comparison (n = 12). Scale bars: 5,0 mm in **a**, 1,0 mm in **b**.

(A)

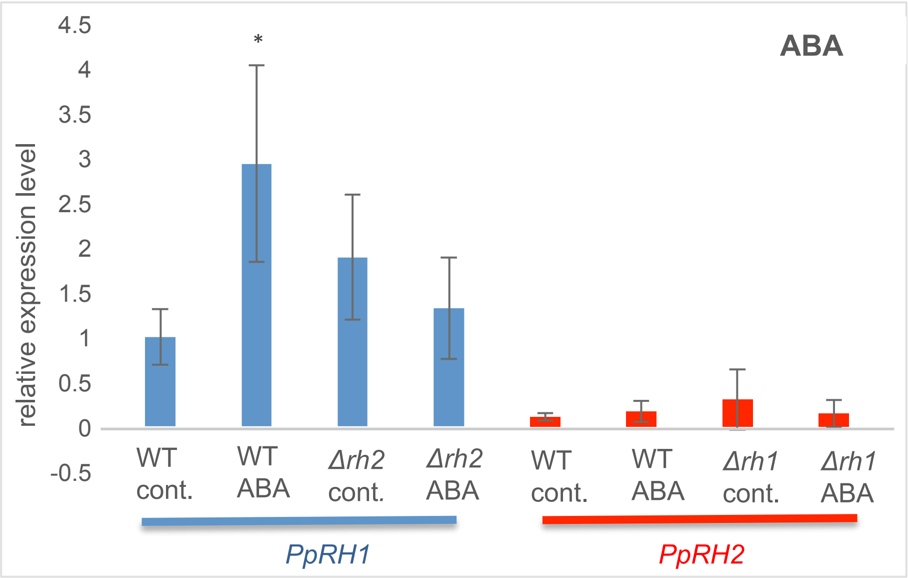

(B)

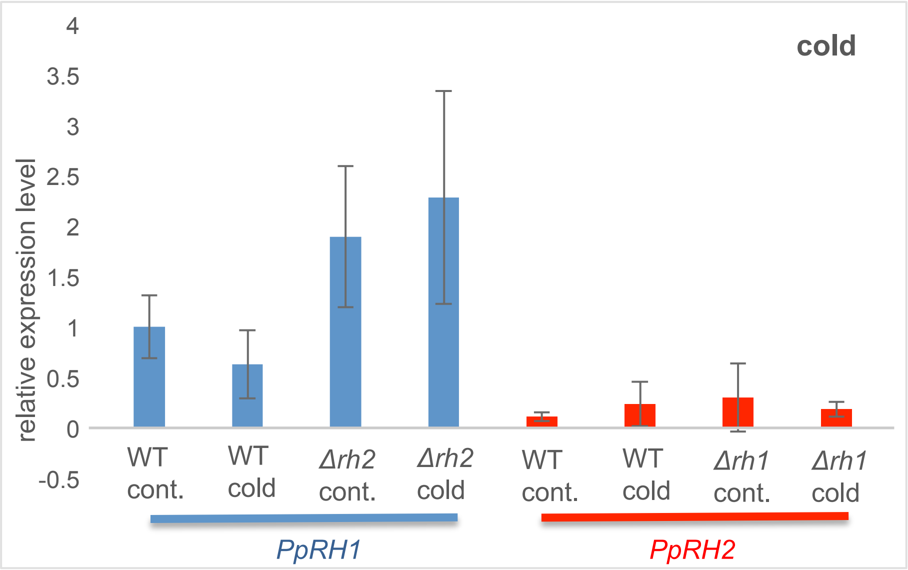

Fig S10 Quantitative PCR analysis of *PpRH1* and *PpRH2* after ABA and cold treatment. **A** Blue bars on the left represent *PpRH1* relative expression level in WT and *Δrh2* mutant under standard conditions (cont.) and 24h treatment on media supplemented with 10 μM ABA. Red bars on the right represent *PpRH2* relative expression level in WT and *Δrh1* mutant under standard conditions (cont.) and 24h treatment on media supplemented with 10 μM ABA. **B** Blue bars on the left represent *PpRH1* relative expression level in WT and *Δrh2* mutant under standard conditions (cont.) and 24h cold treatment (10°C). Red bars on the right represent *PpRH2* relative expression level in WT and *Δrh1* mutant under standard conditions (cont.) and 24h cold treatment. Statistically significant differences between WT at control conditions and treatments from three independent cultures were analysed by Student's *t* test. The statistical significance was attributed at the 0.01 probability level and is denoted as *P < 0.01.

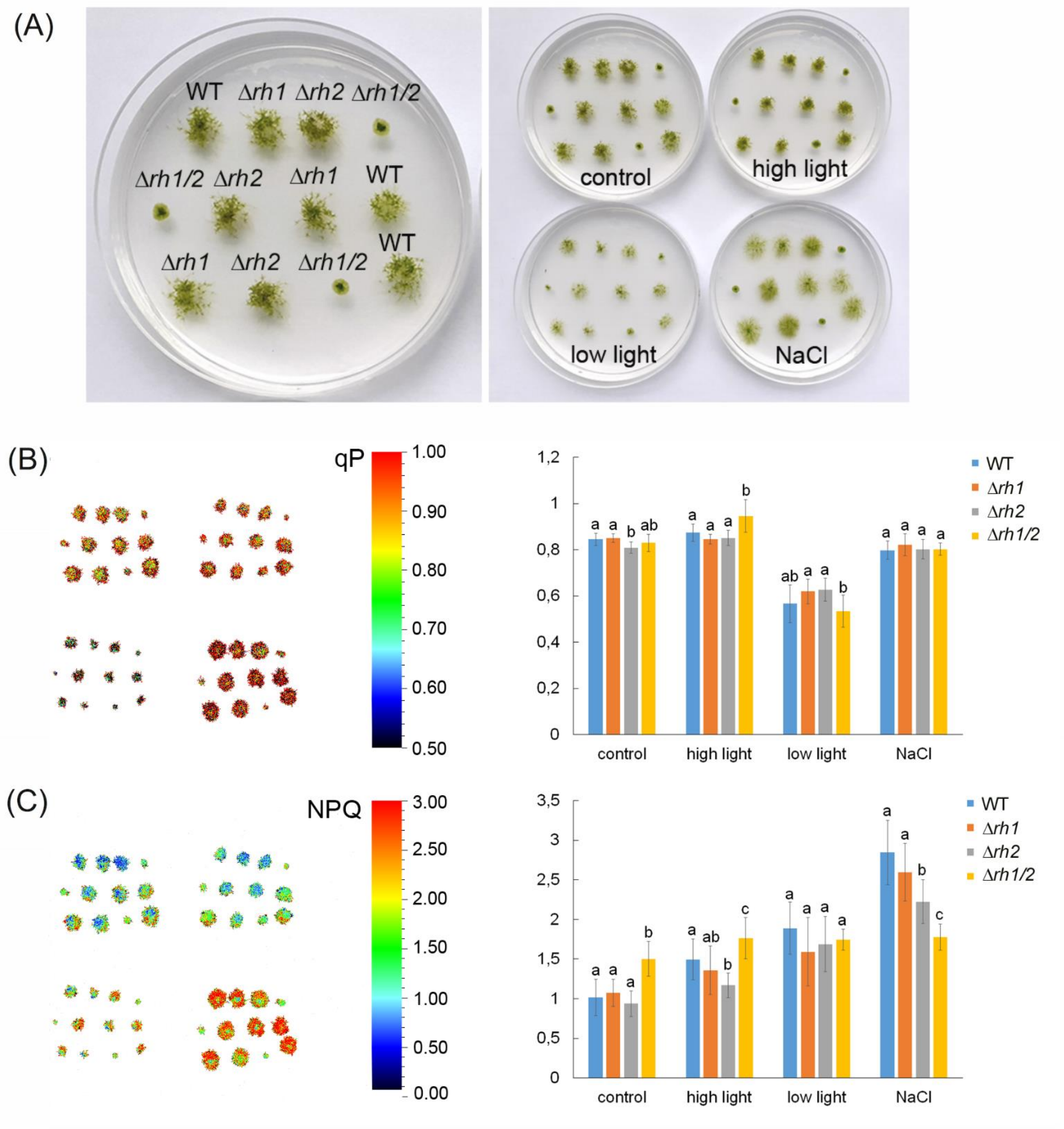

Fig S11 Chlorophyll fluorescence imaging and quantification. a Plant distribution on Petri dish (left) and Petri dishes with individual treatments. **b** Photochemical quenching (qP).

c Non-photochemical quenching (NPQ). On the left, representative images with scales are shown. Plants and plates distribution matches images on **a.** On the right, mean values ± S.D. (n = 12) are shown. Different letters indicate significant differences at $P < 0.05$ within one treatment (ANOVA, Tukey-test).

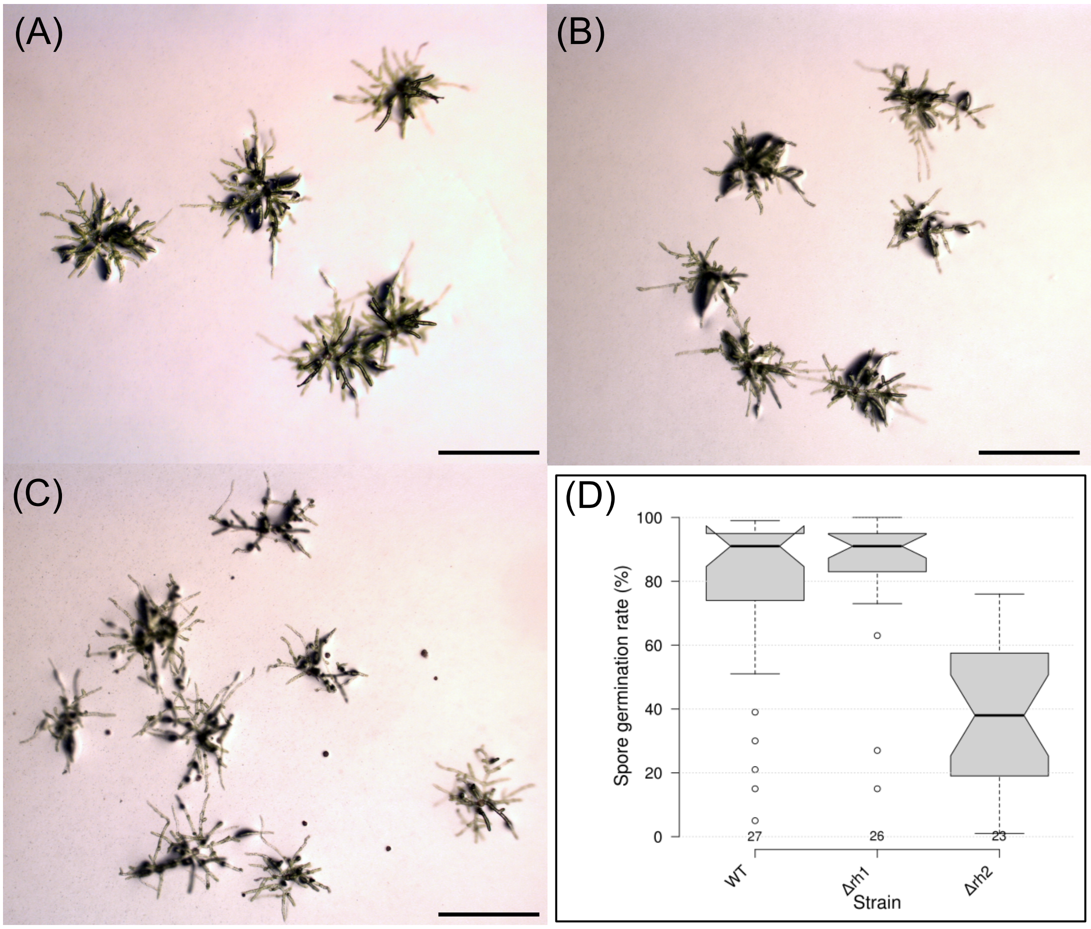

Fig S12 *Δrh2* spores display a reduced germination rate compared to WT and *Δrh1*. Spores were set on BCDA medium and let germinate and grow unto sporelings for seven days under standard growth conditions. **a** Wild-type sporelings. **b** *Δrh1* sporelings. **c** *Δrh2* sporelings. Bar: 500 µm. **d** Box plot displaying spore germination rates of individual sporophyte. Germination rates were assessed with a sample of 100-300 spores per sporophyte. Center lines show the medians; box limits indicate the 25th and 75th percentiles as determined by R software; whiskers extend 1.5 times the interquartile range from the 25th and 75th percentiles, outliers are represented by dots. n = 27, 26, 23 tested sporophytes. The plot was generated using the online tool BoxPlotR: a web-tool for generation of box plots (http://shiny.chemgrid.org/boxplotr/).

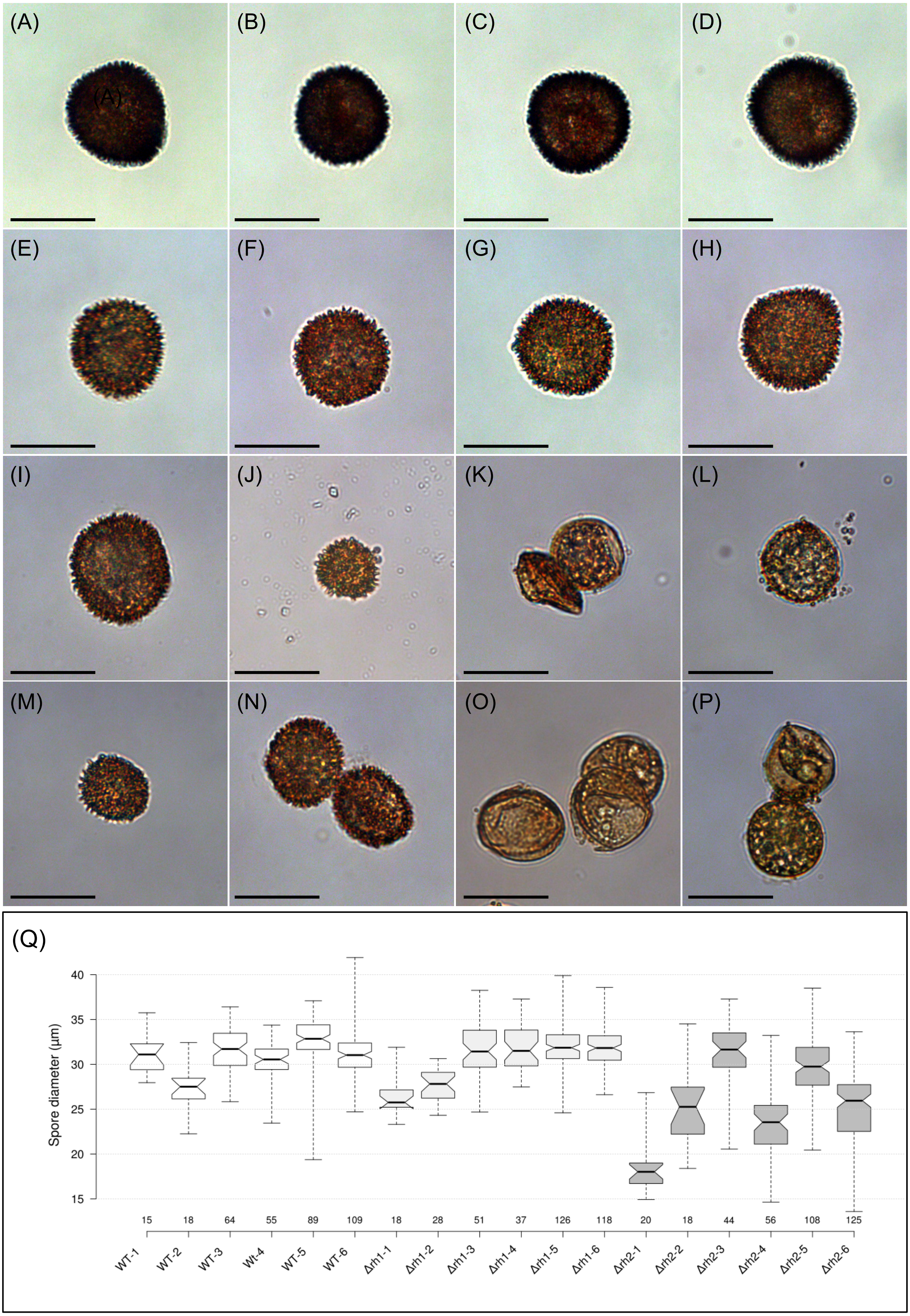
(A)
(A)
(B)
(C)
(D)
(E)
(F)
(G)
(H)
(I)
(J)
(K)
(L)
(M)
(N)
(O)
(P)
(Q)
Spore diameter (µm)
40
35
30
25
20
15
15
18
64
55
89
109
18
28
51
37
126
118
20
18
44
56
108
125
WT-1
WT-2
WT-3
Wt-4
WT-5
WT-6
Δrh1-1
Δrh1-2
Δrh1-3
Δrh1-4
Δrh1-5
Δrh1-6
Δrh2-1
Δrh2-2
Δrh2-3
Δrh2-4
Δrh2-5
Δrh2-6

Fig S13 Mature *Δrh2* spores display a heterogeneous shape and size compared to WT and *Δrh1* spores. a-d: wild type spores. **e-h:** *Δrh1* spores. **i-p**: *Δrh2* spores. Bar: 25 µm. **q**: Box plot diagram displaying the spore diameter distribution for six independent sporophytes of each strain. Center lines shows the diameter median value; box limits indicates the 25th and 75 percentiles as determined by R software, whiskers extend to minimum and maximum value. n value for each spore set is indicated along the y axis. The plot was generated using the online tool BoxPlotR: a web-tool for generation of box plots (http://shiny.chemgrid.org/boxplotr/).

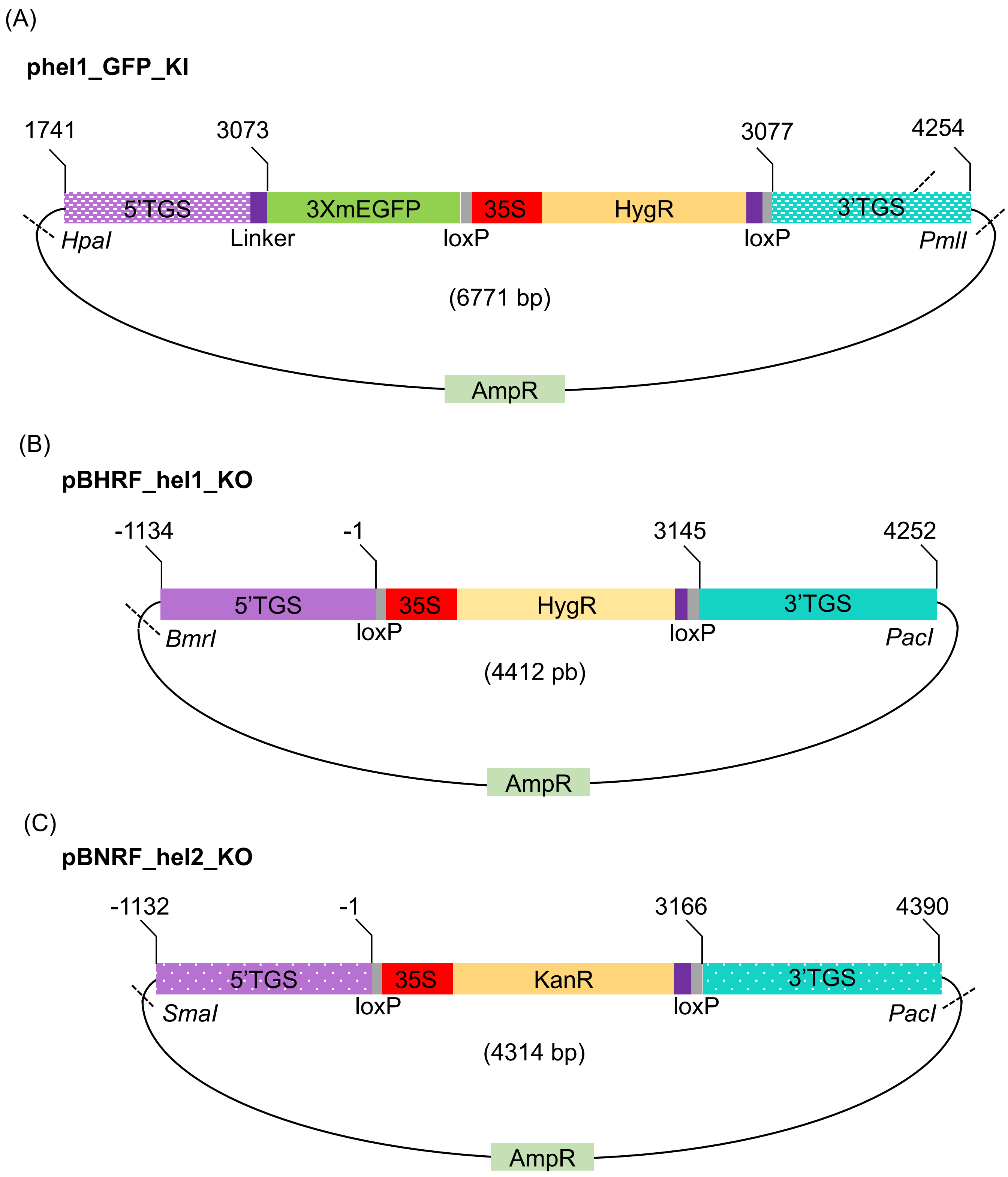

Fig S14 Cartoon representation of the knockout and knock-in plasmid vectors. Plasmids **a** phel1_GFP_KI, **b** pBHRF_hel1_KO and **c** pBNRF_hel2_KO were constructed for the purpose of this study. The numbering corresponds to the nucleotide position relative to the A^{1}TG start site in Pp3c20_20710 (**a** and **b**) and Pp3c10_20840 (**c**) genomic sequences. The restriction enzymes used to linearize the plasmids prior to *P. patens* transformation are indicated with dashed lines. The base pair (bp) numbers in brackets correspond to the sizes of the resulting double restriction-digest fragment. TGS = targeting sequence. Figure not drawn to scale.
